# Nanoparticle-mediated Photodynamic Therapy as a Method to Ablate Oral Cavity Squamous Cell Carcinoma in Preclinical Models

**DOI:** 10.1158/2767-9764.CRC-23-0269

**Published:** 2024-03-15

**Authors:** Axel Sahovaler, Michael S. Valic, Jason L. Townson, Harley H.L. Chan, Mark Zheng, Sharon Tzelnick, Tiziana Mondello, Alon Pener-Tessler, Donovan Eu, Abdullah El-Sayes, Lili Ding, Juan Chen, Catriona M. Douglas, Robert Weersink, Nidal Muhanna, Gang Zheng, Jonathan C. Irish

**Affiliations:** 1 Department of Otolaryngology–Head and Neck Surgery, University of Toronto, Toronto, Ontario, Canada.; 2 TECHNA Institute, Guided Therapeutics (GTx) Program, University Health Network, Toronto, Ontario, Canada.; 3 Princess Margaret Cancer Centre, University Health Network, Toronto, Ontario, Canada.; 4 Institute of Biomedical Engineering (BME), University of Toronto, Toronto, Ontario, Canada.; 5 Department of Otolaryngology–Head and Neck Surgery, Queen Elizabeth University Hospital, Glasgow, United Kingdom.; 6 Department of Medical Biophysics, University of Toronto, Toronto, Ontario, Canada.; 7 Department of Otolaryngology–Head and Neck Surgery, Tel Aviv Sourasky Medical Centre, Tel Aviv University, Tel Aviv, Israel.

## Abstract

Photodynamic therapy (PDT) is a tissue ablation technique able to selectively target tumor cells by activating the cytotoxicity of photosensitizer dyes with light. PDT is nonsurgical and tissue sparing, two advantages for treatments in anatomically complex disease sites such as the oral cavity. We have previously developed PORPHYSOME (PS) nanoparticles assembled from chlorin photosensitizer–containing building blocks (∼94,000 photosensitizers per particle) and capable of potent PDT. In this study, we demonstrate the selective uptake and curative tumor ablation of PS-enabled PDT in three preclinical models of oral cavity squamous cell carcinoma (OCSCC): biologically relevant subcutaneous Cal-33 (cell line) and MOC22 (syngeneic) mouse models, and an anatomically relevant orthotopic VX-2 rabbit model. Tumors selectively uptake PS (10 mg/kg, i.v.) with 6-to 40-fold greater concentration versus muscle 24 hours post-injection. Single PS nanoparticle–mediated PDT (PS-PDT) treatment (100 J/cm^2^, 100 mW/cm^2^) of Cal-33 tumors yielded significant apoptosis in 65.7% of tumor cells. Survival studies following PS-PDT treatments demonstrated 90% (36/40) overall response rate across all three tumor models. Complete tumor response was achieved in 65% of Cal-33 and 91% of MOC22 tumor mouse models 14 days after PS-PDT, and partial responses obtained in 25% and 9% of Cal-33 and MOC22 tumors, respectively. In buccal VX-2 rabbit tumors, combined surface and interstitial PS-PDT (200 J total) yielded complete responses in only 60% of rabbits 6 weeks after a single treatment whereas three repeated weekly treatments with PS-PDT (200 J/week) achieved complete ablation in 100% of tumors. PS-PDT treatments were well tolerated by animals with no treatment-associated toxicities and excellent cosmetic outcomes.

**Significance:**

PS-PDT is a safe and repeatable treatment modality for OCSCC ablation. PS demonstrated tumor selective uptake and PS-PDT treatments achieved reproducible efficacy and effectiveness in multiple tumor models superior to other clinically tested photosensitizer drugs. Cosmetic and functional outcomes were excellent, and no clinically significant treatment-associated toxicities were detected. These results are enabling of window of opportunity trials for fluorescence-guided PS-PDT in patients with early-stage OCSCC scheduled for surgery.

## Introduction

Oral cavity squamous cell carcinoma (OCSCC) is the most prevalent form of head and neck cancer with 377,000 new cases diagnosed globally each year (1). Most OCSCCs arise from the oral tongue, lip, and floor of mouth (2) wherein the standard of care is primarily surgical excision or radiotherapy with external beam or interstitial implantation (3). Advances in tissue conserving surgical treatments such as transoral laser microsurgery and transoral robotics offer patients with early-stage OCSCC significant survival benefits and fewer functional morbidity and long-term toxicity as compared with conventional excisional approaches. The majority of patients with OCSCC present with locally advanced and regional disease (4), requiring combinations of surgical, radiation, and systemic (e.g., platinum-based chemotherapy, immunotherapy) treatment modalities that result in severe side effects reducing patient quality of life. The toxicities and poor functional and aesthetic outcomes of current treatments for advanced stage OCSCC have motivated the search for better treatment options that are safe, function preserving, and repeatable with minimal toxicity.

Photodynamic therapy (PDT) is a nonsurgical treatment modality in which light is used to activate a systemically administered photosensitizer drug (or “dye”) in tissues and generate cytotoxic reactive oxygen species. PDT treatments are highly selective owing to (i) the preferential uptake of photosensitizers in tumor cells versus healthy mucosal cells and (ii) confinement of the cytotoxic PDT effects only to tissues directly illuminated with the appropriate wavelength of light to activate the photosensitizer (ideally >600 nm for deeper penetration in tissues). The therapeutic mechanisms of PDT are oxygen dependent and include combinations of direct apoptotic and/or necrotic tumor cell death, damage to the tumor vasculature, and induction of inflammatory reactions and systemic antitumor immunity [i.e., abscopal effect (5)]. In contrast to surgical resection, PDT treatments in the oral cavity are tissue sparing and can preserve the connective collagen matrix in the treated area (6) upon which surrounding mucosal cells can repopulate and restore tissue function and cosmetic appearance. Aside from tumor hypoxia, there are no known resistance mechanisms in cancer cells to PDT with porphyrin-type photosensitizers (7) and treatments can be repeated multiple times in the same lesion and patient.

Numerous photosensitizer drugs have been investigated in clinical trials for PDT of oral cavity cancers (8). The most extensively studied photosensitizer in OCSCC is mTHPC (Foscan), which has demonstrated complete response rates >65% and low local recurrence rates for early-stage T1/T2 N0 disease (9, 10) comparable to routine transoral surgical treatments (11, 12). Another photosensitizer, porfimer sodium (Photofrin), has similarly achieved complete response rates between 69% and 88% in early-stage OCSCC and disease-specific 5-year survival >80% after PDT (13, 14). In locally advanced T3/T4 stage and recurrent OCSCC disease, mTHPC-PDT exhibited favorable tumor response rates and overall survival for lesions with depth of invasion ≤10 mm, which is about the effective penetration depth of 653-nm light used for activating mTHPC (15, 16). The most common side effects of PDT reported in patients with OCSCC were local and facial pain after treatment, edema, necrosis, and photosensitivity-related reactions (8) due to the background accumulation of drug in the skin and leading to prolonged photosensitization of patients to natural and artificial lighting lasting weeks to months post-administration.

Current PDT research has focused on the development of photosensitizers possessing multifunctional properties for image-guided therapy, enhanced delivery to (and selectivity for) tumor cells, and reduced accumulation and sensitization to off-target tissues (e.g., skin). Toward this end, our group had previously developed PORPHYSOMES (PS), a phototheranostic nanotechnology platform combining capabilities for optical imaging (for treatment planning and guidance) and antitumor phototherapy into a single product (17–19). PS are PEGylated liposomal nanoparticles assembled from multifunctional building blocks consisting of a phospholipid conjugated with a chlorin photosensitizer (a.k.a, porphyrin-lipid conjugate). Nanoparticles are ideal delivery vehicles for photosensitizers by offering high drug payloads and enhanced uptake and retention in pathologic tissues via active transport processes (20, 21).

**FIGURE 1:**
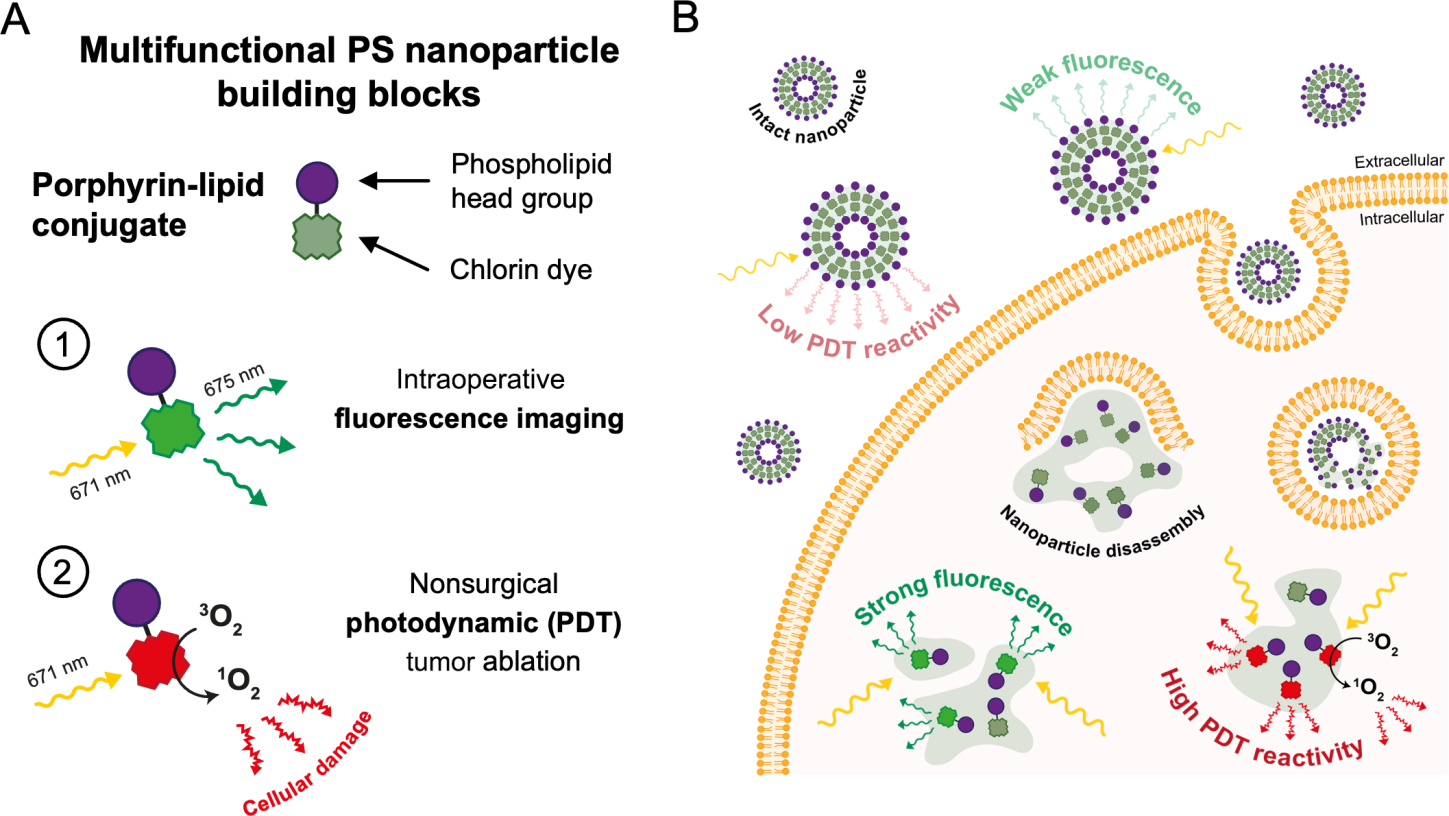
Overview of the phototheranostic applications of PS nanotechnology in cancer surgery. **A,** Porphyrin-lipid conjugate is a multifunctional nanoparticle building block capable of fluorescence image–guided treatment planning and PDT with illumination using 671-nm light. **B,** Structurally driven unquenching (or activation) of porphyrin-lipid conjugate fluorescence and photodynamic reactivity following PS nanoparticle cellular uptake and disassembly.

The multifunctionality of PS nanoparticles arise from the porphyrin-lipid conjugate subunits which possess near infrared (NIR) fluorescence and potent PDT reactivity upon illumination with 671-nm light (Fig. 1A). Unlike conventional molecular photosensitizers like mTHPC or porfimer sodium whose photoactivity is “always on,” the tightly packed arrangement of porphyrin-lipid conjugate within the bilayer of intact PS particles quenches their fluorescence and singlet oxygen generation. Consequently, accumulation of PS in off-target tissues such as healthy mucosa or skin does not lead to their immediate photosensitization (19). Upon disassembly of the PS nanostructure following uptake into cells, the porphyrin-lipid conjugates unquench and restore their fluorescence signal and PDT reactivity (Fig. 1B). Therefore, the structurally driven activation of PS uniquely enhances the selectively of PDT effects to tissues and cell types that avidly uptake nanomaterials while sparing surrounding healthy tissues and cells in which PS nanoparticles remain predominantly intact and quenched.

With the aim of building upon the clinical successes of PDT in OCSCC and enhancing treatment multifunctionality and selectivity over existing clinically tested photosensitizer drugs, we describe herein the development of PS nanoparticle–mediated PDT (PS-PDT) as a safe and repeatable nonsurgical tumor ablation strategy in OCSCC. Using multispecies models of oral cavity cancer with varying biological and anatomic relevance to human disease, we confirmed the selective accumulation and activation of PS in squamous cell tumors using fluorescence imaging and microscopy. We analyzed how the intratumoral distribution of PS impacted responses to PDT on a cellular level. And finally, in therapeutic efficacy studies, the safety, clinical response, and functional and cosmetic outcomes following single or repeated PS-PDT treatments in three tumor models were evaluated.

## Materials and Methods

Methodologic details for the preparation and physicochemical characterization of PS nanoparticles, plasma and tissue pharmacokinetic analysis in animal models of squamous cell oral cavity cancer, histologic tissue preparation and quantitative analysis, and PS safety assessments are provided in Supplementary Materials and Methods.

### Review Board Approvals for Animal Studies

Animal studies were conducted at the Animal Resource Centre of the University Health Network in accordance with protocols #6308 (mice) and #2931 (rabbits) approved by the Ontario Cancer Institute Institutional Animal Care Committee.

### *In Vivo* Models of Squamous Cell Oral Cavity Cancer

#### Subcutaneous Tumor-bearing Mouse Models

Centre Antoine Lacassagne-33 (Cal-33) cells (DSZM ACC 447) derived from a human tongue squamous cell carcinoma (22) were cultured in 90% DMEM (Gibco 11966025) supplemented with 10% FBS (Gibco 12483020). Mouse oral squamous cell carcinoma (MOC22) cells (Kerafast EWL003-FP) derived from primary tumors in C57BL/6 wild-type mice (23) were cultured in 90% 2:1 Iscove's DMEM (Gibco 12440053) to Ham's F-12K (Gibco 21127022) media supplemented with 10% FBS. Authentication testing of Cal-33 and MOC22 cell lines were completed and documented by the suppliers and *Mycoplasma* testing completed by PCR annually. Immunocompromised 8–10 weeks old female athymic nude mice (Envigo 069) were injected subcutaneously in the right flank with 3 × 10^6^ Cal-33 cells (passage number < 4) using a 27 Ga syringe. Immune competent 8–10 weeks old female C57BL/6J mice (The Jackson Laboratory 000664) were injected subcutaneously in the right flank with 5 × 10^6^ MOC22 cells (passage number < 4) using a 27 Ga syringe. Cal-33 xenograft and MOC22 syngeneic tumor models were obtained within 1–2 weeks of cell implantation.

#### Orthotopic Tumor-bearing Rabbit Model

Leporine-derived virus-induced anaplastic squamous cell carcinoma (VX-2) cells (24) were obtained from frozen tumor tissue previously propagated in the thigh of a rabbit (passage number not applicable). To obtain a single-cell suspension, the tumor pieces were thawed, minced, and passed through a 70-mm cell strainer (Corning C352350) using Hanks’ Balanced Salt Solution (HBBS; Sigma-Aldrich H9394). Authentication and *Mycoplasma* testing of VX-2 cells was not performed. Approximately 1.5 × 10^6^ VX-2 cells in a 300 µL volume of HBBS were injected into the buccinator muscle (buccal area) of anesthetized 3.0 kg male New Zealand white rabbits (Charles River) to obtain an orthotopic, anatomically relevant OCSCC model.

### PS-PDT Treatment in Tumor-bearing Mice

PS nanoparticles were intravenously administered to mice (∼0.020 kg body weight [BW]) at a dose of 10 mg/kg diluted in 0.9% sodium chloride injection (Baxter 1301). Administration was an intravenous bolus via the tail vein using a 31 Ga needle. Twenty-four hours post-injection mice were sedated with inhaled anesthesia and fur overlaying the subcutaneous tumor removed using a depilatory agent. The subcutaneous tumors were surfaced illuminated through the overlying skin using a 671-nm diode pumped solid state (DPSS) laser (LaserGlow Technologies R6710B1FX) and a cut-end fiber with a 10 mm beam diameter. Each tumor received 100 J/cm^2^ light fluence at 100 mW/cm^2^ fluence rate. Following treatment mice were administered buprenorphine (0.1 mg/kg, subcutaneous) as needed for pain management and returned to normal grouped housing.

### Histologic Evaluation and Quantitative Analysis of PS-PDT Photodamage

Subcutaneous Cal-33 xenograft tumor-bearing mice were administered PS (10 mg/kg, i.v.) via tail vein injection followed by PDT treatment 24 hours post-injection. The tumors were externally irradiated using a 671-nm DPSS laser and a cut-end fiber with a 9-mm diameter for a fluence of 100 J/cm^2^ (100 mW/cm^2^ fluence rate). Mice receiving no treatment/intervention (untreated control), receiving PS administration only (drug control), or surface PDT laser illumination only (light control) were also included as treatment groups. Three days following PS-PDT or control treatments, the animals were euthanized and the tumors excised for histopathologic analysis for photodamage. Partially responding tumors to PS-PDT treatment after 14 days were also analyzed. Tumor tissues were fixed in formalin solution (Sigma-Aldrich HT501128), paraffin embedded, and tissue blocks serially sectioned with slice thickness of 4 µm. Staining with hematoxylin and eosin (H&E) was performed as per laboratory standard operating procedures. Terminal deoxynucleotidyl transferase dUTP nick end labeling (TUNEL) was performed according to the suppliers’ protocol (Roche 03333566001, Roche 11093070910) and was visualized using 3,3′-Diaminobenzidine (DAB) substrate (Abcam ab64238). IHC staining with anti-cleaved caspase-3 (Cell Signaling Technology 9661) was performed according to the suppliers’ protocol and counterstained with biotinylated anti-rabbit IgG (Vector Laboratories BA-1000-1.5) and DAB substrate according to the suppliers’ protocols. Whole slide brightfield scans of H&E, TUNEL, and cleaved caspase-3 stained tumor sections were acquired with 20x resolution.

Quantification of cleaved caspase-3 staining in tumor histology from the PS-PDT treated group (day 3 and day 14) and untreated, drug, and light control groups was performed using histologic image analysis software (Indica Labs HALO). A cellular classifier was created using a random forest machine learning algorithm to classify different types of tissues/cells within the tumor microenvironment (e.g., whitespace, necrosis, tumor, healthy tissues) based on the image color and texture from provided examples. Analysis output consisted of the areas of the different tissue types, total number of cells, and the percentage of cleaved caspase-3 positive cells within each tissue. The percentage (%) of classifier-identified tumor cells positive for cleaved caspase-3 staining was calculated, and the mean ± SD for the PS-PDT treated (day 3 and day 14), and untreated, drug, and light control groups (*N* = 2–3 tumors analyzed/treatment group) were reported.

### Single and Repeat PS-PDT Treatment in VX-2 Tumor-bearing Rabbits

PS nanoparticles were intravenously administered to tumor-bearing rabbits (∼3.5 kg BW) at a dose of 10 mg/kg diluted in 0.9% sodium chloride. Administration was a short intravenous infusion over 5 minutes via the marginal ear vein. Twenty-four hours post-injection rabbits were sedated with inhaled anesthesia and fur overlying the orthotopic buccal tumor removed using a depilatory agent. PDT treatment of tumors were performed in two sequential steps: step 1, surface illumination of tumor through the overlying skin using a cut-end fiber with a 10-mm beam diameter, and step 2, interstitial illumination of the tumor with a 1-cm cylindrical light diffusing fiber optic (Medlight RD-ML10) inserted transcutaneous under ultrasound guidance. A 671-nm DPSS laser was used for both steps delivering a total light fluence of 200 J (100 J per step) at a fluence rate of 100 mW/cm^2^ (in step 1) or mW/cm (in step 2). Following treatment, rabbits were administered buprenorphine (0.05 mg/kg, i.v. or subcutaneous) as needed for pain management and returned to normal housing. If the clinical response following the initial PS-PDT treatment was deemed suboptimal, two additional PS-PDT treatments were repeated weekly for the same tumor with identical parameters described above for a total of three PS-PDT treatments per tumor and rabbit.

### PS-PDT Treatment Efficacy Models

#### Definitions

Tumor responses were classified as per World Health Organization criteria (25). Complete response was defined as no measurable nor palpable tumor mass. Partial response was defined as 50% or more decrease in measurable tumor volume compared with the time of treatment (i.e., day 0 or week 0). No response/no change was defined as decrease of less than 50% or less than 25% increase in measurable tumor volume compared with the time of treatment. Treatment failure (or progressive disease) was defined as 25% or more increase in measurable tumor volume compared with the time of treatment.

#### Tumor-bearing Mouse Models

Fifty-three subcutaneous Cal-33 xenograft tumors were grown until tumor volume reached approximately 100 mm^3^ (or ≤7 mm in greatest dimension) and then randomized into four treatment groups: 20 PS-PDT, 17 untreated controls, nine drug controls (PS administration only), and seven light controls (illumination only). Twenty-four subcutaneous syngeneic MOC22 tumors were grown until tumor volume reached approximately 65 mm^3^ (or 5 mm in greatest dimension) and randomized into two treatment groups: 11 PS-PDT and 13 untreated controls. Following surface PS-PDT or control treatments, mice were monitored daily for 14 days and tumor size tracked with calliper measurements on days 3, 7, and 14 post-treatment. Tumor volume was calculated according to formulae π/6(*L* × *W*^2^) where *L* is the long axis and *W* is the short axis. Humane endpoint was tumor volume ≥1.50 cm^3^. Residual tumor tissue from PS-PDT treated mice were submitted for histopathologic analysis on the day 14 endpoint as needed.

#### VX-2 Tumor-bearing Rabbits

Sixteen orthotopic syngeneic VX-2 tumor models were grown up to at least approximately 524 mm^3^ (or 10 mm in greatest dimension) and randomized into four treatment groups: five single PS-PDT, four repeat PS-PDT, three drug controls, and three light controls. Single PS-PDT treatment animals underwent the “two-step” PDT treatment once, whereas repeat PS-PDT treatment animals received three repeated weekly PDT treatments for the same tumor. Light control animals received “two-step” light irradiation only and rabbits in drug control group received PS administration only. Following treatment, rabbits were monitored biweekly and tumor size measured with CT imaging weekly for up to 6 weeks after start of treatment. Region of interest measurements on CT were used to determine tumor volume. Humane endpoints included tumor volume ≥3.05 cm^3^. Residual tumor tissue from the single and repeat PS-PDT treated rabbits were submitted for histopathologic analysis on the week 6 endpoint as needed.

### Statistical Analysis

Parameter mean ± SD is reported in text. Pre-experimental power analysis was not performed for *in vivo* efficacy and survival studies. For analyzing changes in tumor volume on day 14 posttreatment, multiple unpaired (two-sample) *t* tests using Holm-Šídák correction method for multiple comparisons was used. For analyzing changes in hematology parameters, unpaired *t* tests without correction for multiple comparisons was used. For analyzing survival curves, multiple comparisons with log-rank tests using Bonferroni correction method for multiple comparisons were used. Significance level ⍺ = 0.05 was used throughout and *P* values reported using NEJM reporting style: * <0.05, ** <0.01, *** <0.001. Statistical testing and data graphing were performed with Prism 9 (GraphPad Software Version 10.1.0).

### Data Availability Statement

The data generated in this study is available upon request.

## Results

### Physicochemical Characterization of PS

**FIGURE 2:**
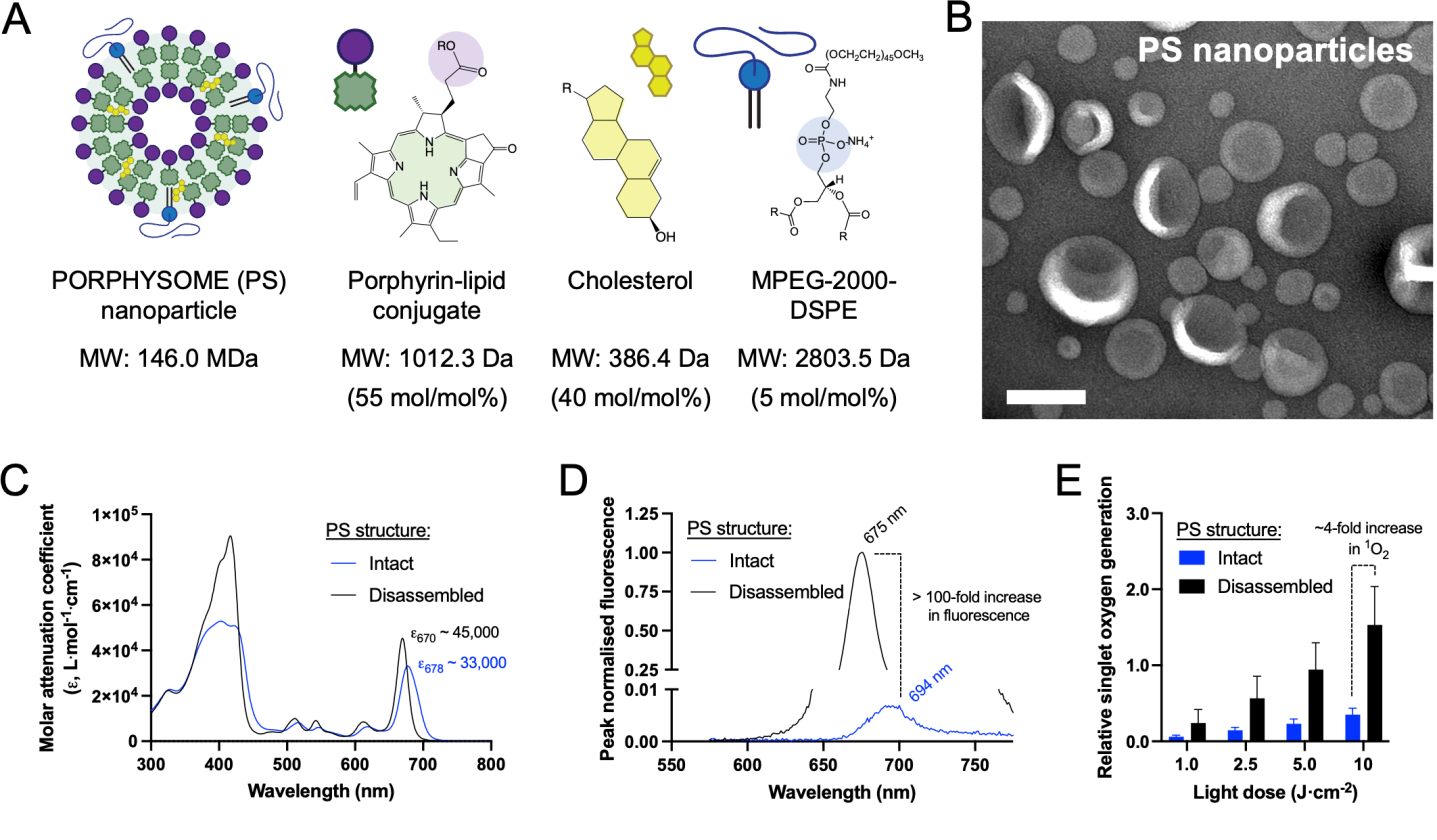
Photophysical and photochemical characterization of PS nanoparticles. **A,** Structure and composition of PS: PEGylated porphyrin-lipid conjugate-containing nanoparticles. **B,** Transmission electron microscopy of negatively stained PS nanoparticle morphology. Scale bar, 0.1 µm. **C,** Molar attenuation coefficient (ε) profile of intact and disassembled PS in aqueous media. Intact PS are suspended in 1x PBS and disassembled PS are treated with a non-ionic surfactant (Triton X-100). Note ε reported here on basis of mols of porphyrin-lipid conjugate. **D,** Fluorescence spectra of intact and disassembled PS nanoparticles in aqueous media at excitation wavelength 416 nm. Note the 100-fold increase in fluorescence intensity in the disassembled PS versus the intact PS at 675-nm wavelength. **E,** Singlet oxygen (^1^O_2_) generation of intact and disassembled PS nanoparticles in aqueous media with increasing light dose. Note the approximately 4-fold increase in ^1^O_2_ generation in the disassembled PS versus the intact PS using 671-nm excitation wavelength (50 mW). Mean ± SD. *N* = 5 samples/light dose. Additional material characterizations of PS described in Supplementary Fig. S1 and Supplementary Table S1.

PS form spherical nanovesicles with a unilamellar bilayer surrounding an aqueous core, a similar morphology to conventional PEGylated liposomes (Fig. 2A and B). On the basis of their measured molecular weight of 146.0 ± 9.9 MDa, each PS nanoparticle contains approximately 94,000 photosensitizers on average (Supplementary Table S1). Both the intact PS and disassembled porphyrin-lipid conjugate monomer demonstrated strong absorption around 670-nm wavelength with a molar extinction coefficient of approximately 45,000 L/mol·cm for the disassembled monomer (Fig. 2C). The structurally driven activation of PS nanoparticle fluorescence signal and photoactivity was confirmed by the 100-fold increase in fluorescence (Fig. 2D) and 4-fold increase in cytotoxic single oxygen generation (Fig. 2E) from PS disassembled with aqueous surfactants versus intact PS. Radiolabeling PS with low activity levels of positron-emitting Copper-64 (^64^Cu) permitted highly sensitive radiotracing of PS organ pharmacokinetics and tissue uptake *in vivo* without affecting the physicochemical properties of the nanoparticle (see Supplementary Fig. S1).

### Pharmacokinetics of PS in Preclinical Models of Oral Cavity Cancer

**FIGURE 3:**
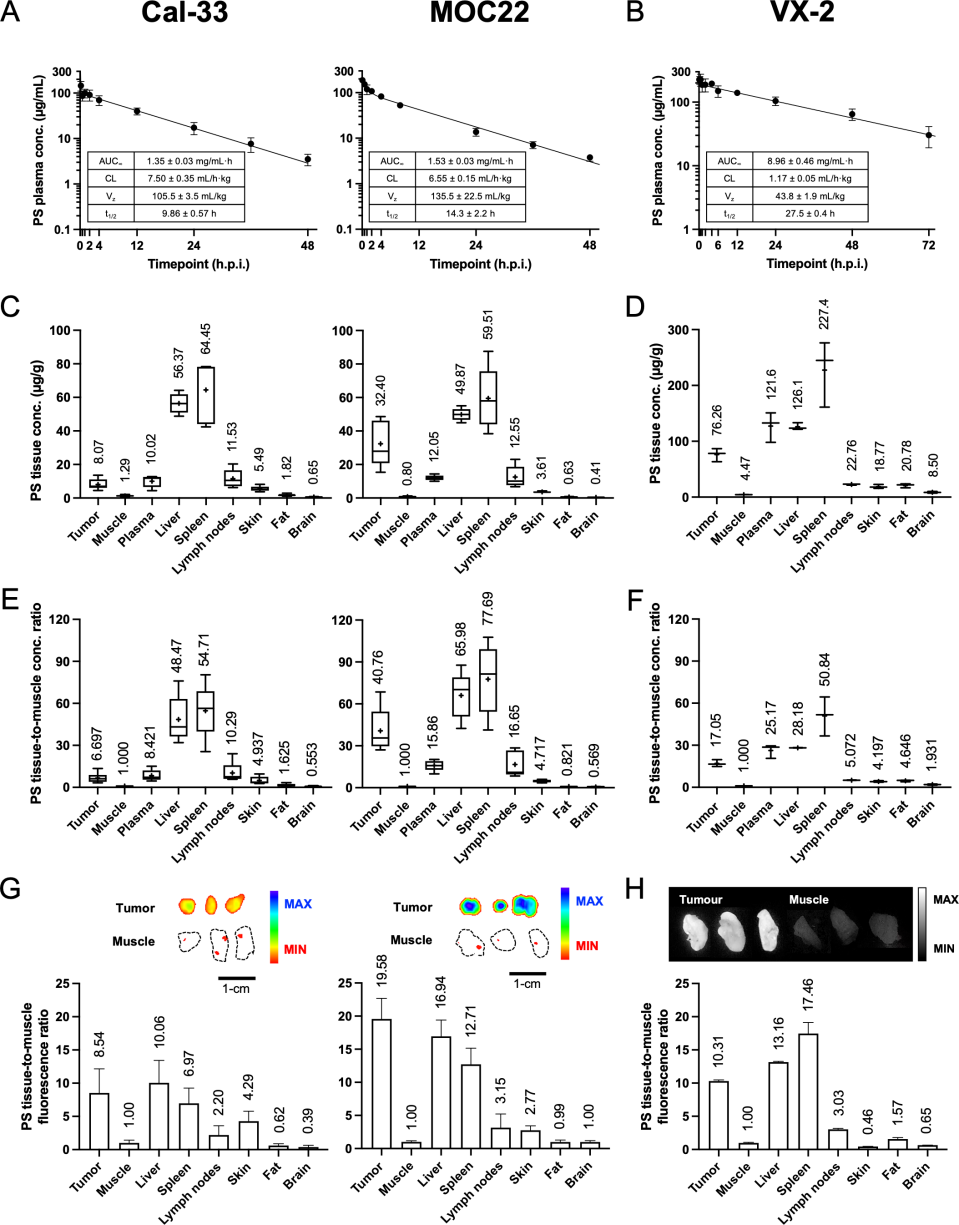
Pharmacokinetic profiles of PS nanoparticles in tumor-bearing mice and rabbit models of oral cavity cancer. Plasma concentration-time profiles of PS in Cal-33 and MOC22 mouse models (**A**) and VX-2 rabbit model (**B**). Units µg/mL. Additional pharmacokinetic analysis is detailed in Supplementary Table S2. Tissue concentrations of PS in Cal-33 and MOC22 mouse models (**C**) and VX-2 rabbit model (**D**) 24 hours post-injection. Units µg/g. Additional tissue distribution data are provided in Supplementary Tables S3–S5. Ratios of PS tissue concentration normalized to muscle concentration in Cal-33 and MOC22 mouse models (**E**) and VX-2 rabbit model (**F**) 24 hours post-injection. Unitless. Representative fluorescence images and ratios of PS *ex vivo* tissue fluorescence normalized to muscle fluorescence in Cal-33 and MOC22 mouse models (**G**), and VX-2 rabbit model (**H**) 24 hours postinjection. Unitless. Additional PS tissue selectivity data are provided in Supplementary Tables S6 and S7. For A, B, Mean ± SD. Table statistics: mean ± SE *N* = 5 mice/model. *N* = 6 rabbits. For C, D, E, and F, Tukey box-and-whisker plot with “+” denoting mean (mean value labeled above). *N* = 5 mice/tissue/model. *N* = 3 rabbits/tissue. For G and H, Bar plot with mean + SD (mean value labeled above). *N* = 5 mice/tissue/model. *N* = 3 rabbits/tissue. For **G**, Ex: 675 nm, Em: 720 nm, 1 second exposure time. For **H**, Ex: 675 nm, Em: 720 nm long pass. For mouse models: PS dose = 10 mg/kg, 400–500 MBq ^64^Cu/kg, i.v. For rabbit models: PS dose = 10 mg/kg, i.v.

The pharmacokinetics of PS nanoparticles was evaluated in Cal-33 and MOC22 mouse models (Fig. 3A) and VX-2 rabbit models (Fig. 3B) following intravenous injection. The plasma concentration-time curve of PS exhibited a classical “two-compartment” profile, consisting of a short distribution phase lasting minutes followed by a slow elimination phase lasting hours. The pharmacokinetic parameters of PS in plasma were consistent between the two mouse models and along with the profile in rabbits were comparable to values for PEGylated nanoparticles of similar size and surface charge (see Supplementary Table S2; refs. 26, 27). Notably, the elimination phase of PS in VX-2 rabbits was significantly longer lasting 27.5 ± 0.4 hours compared with 9.86 ± 0.57 hours and 14.3 ± 2.2 hours in the Cal-33 and MOC22 mouse models, respectively.

The tissue distribution of PS nanoparticles was evaluated in the three tumor models 24 hours post-injection (Fig. 3C and D). PS exhibited significant accumulation in the organs of the mononuclear phagocyte system including the liver and spleen, a distribution pattern characteristic of PEGylated nanoparticles in rodents following intravenous administration (see Supplementary Fig. S2; ref. 28). Notable differences in PS concentration were observed between the human-derived Cal-33 xenograft tumor (8.07 ± 3.11 µg/g) versus the syngeneic MOC22 tumor (31.40 ± 13.61 µg/g) for equivalent dose and timepoint. In other organs, the tissue distribution of PS was comparable between the two mouse models (Supplementary Tables S3 and S4). In the VX-2 rabbit tumors, the concentration of PS was 76.26 ± 12.01 µg/g 24 hours post-injection. Higher absolute and relative PS concentrations were also measured in the other rabbit organs compared with mice at an equivalent mg/kg drug dose (Supplementary Table S5), likely owing to the longer circulatory half-life of PS in rabbits. The selectivity of PS accumulation in the tumors was assessed from the ratio of tumor concentration-to-muscle concentration (T/M; as in Fig. 3E and F): the T/M ratios 24 hours post-administration were 6.70 ± 1.00 and 40.76 ± 16.30 in the Cal-33 and MOC22 models respectively, and 17.05 ± 2.80 in the VX-2 model (Supplementary Table S6).

Finally, confirmation of PS nanoparticle disassembly in the tumors was performed with *ex vivo* tissue fluorescence imaging. As illustrated in Fig. 3G and H, significant PS fluorescence signal was detected in all three tumor models 24 hours after administration whereas the muscles exhibited negligible fluorescence signal. The relative fluorescence in the tumors versus muscle were 8.54 ± 3.63 and 19.58 ± 3.09 in the Cal-33 and MOC22 mouse models, respectively, and 10.31 ± 0.16 in the VX-2 rabbit model (Supplementary Table S7), in agreement with the T/M ratios measured from nanoparticle accumulation in Fig. 3E and F. In fact, the broad similarities between tissue-to-muscle ratios measured with tissue fluorescence and tissue concentration across multiple organs (see Supplementary Fig. S3) implies that PS fluorescence imaging can be a reliable indicator of relative PS tissue uptake *in vivo*. Altogether the fluorescence imaging data confirm that disassembly of PS in the tumor was highly efficient after 24 hours *in vivo*.

### Histologic Evaluation of Intratumoral PS Distribution and PS-PDT Damage in Mouse Models of Oral Cavity Cancer

**FIGURE 4:**
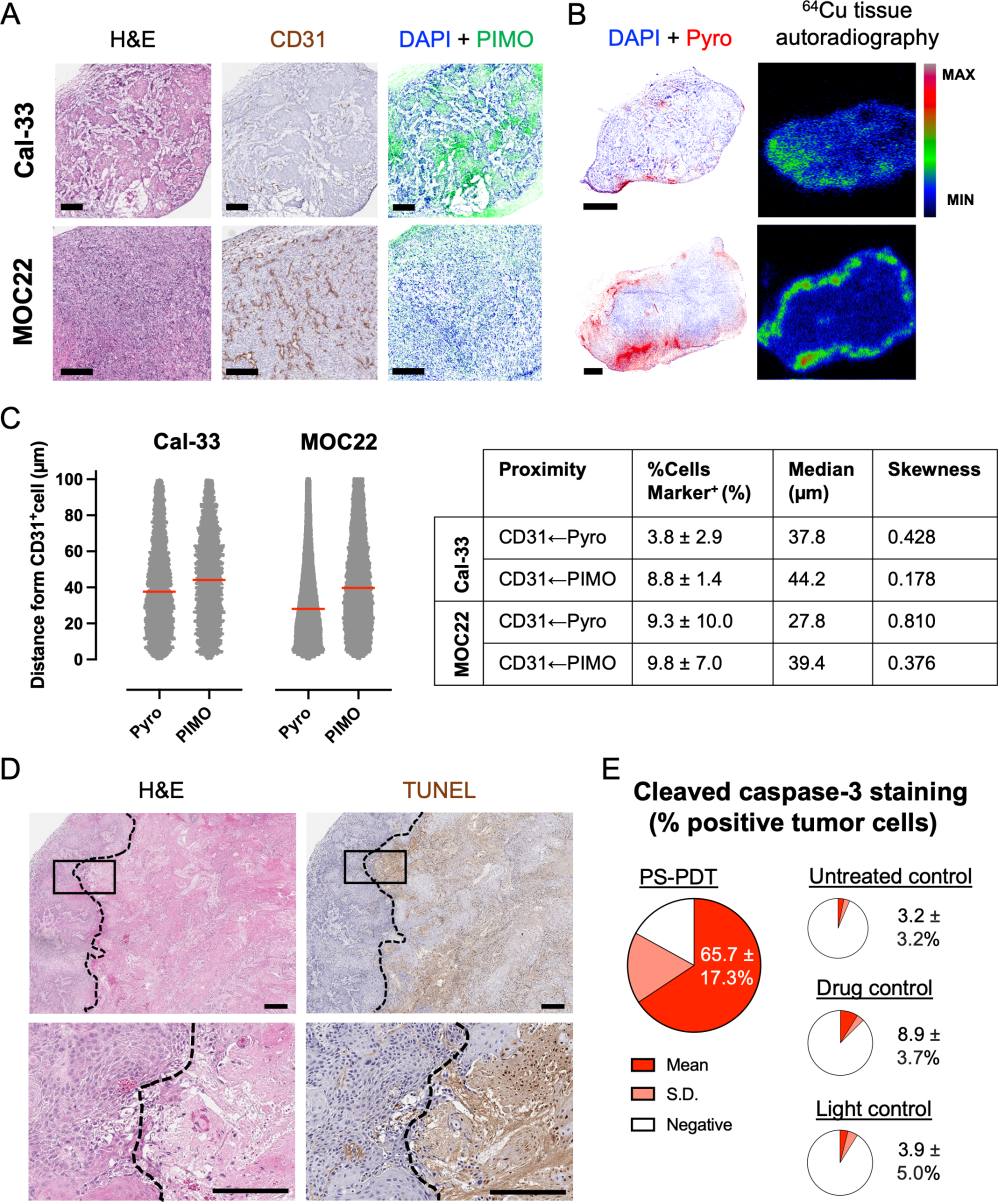
Histologic examination of intratumoral PS nanoparticle accumulation and mechanisms of PS-PDT damage in tumor-bearing mice models of oral cavity cancer. **A,** Brightfield images of the pathophysiology of Cal-33 xenograft tumors (top) and syngeneic MOC22 tumors (bottom). Scale bars = 250 µm. **B,** Imaging of PS nanoparticle intratumoral distribution using fluorescence microscopy (PS signal labeled “Pyro”) and tissue autoradiography of ^64^Cu-labeled PS radioactivity in Cal-33 (top) and MOC22 (bottom) tumors. Scale bars = 1,000 µm. Note that labeling PS with ^64^Cu permit radiotracing of PS pharmacokinetics in tissues and cells using radioactivity (Supplementary Fig. S1). Additional histologic images of other mouse organs are provided in Supplementary Fig. S4. **C,** Quantitative spatial analysis of cellular proximity of Pyro^+^ cells to CD31^+^ cells (CD31←Pyro) and of PIMO^+^ cells to CD31^+^ cells (CD31←PIMO) in Cal-33 and MOC22 tumors. Units µm. Scatter plot of individual replicates (●) with median distance identified by red line. *N* = 4–5 tumors/model. Additional quantitative intratumoral spatial analysis data provided in Supplementary Fig. S5. **D**, Histologic evidence of antitumor response in a Cal-33 xenograft tumor 72 hours after PS-PDT treatment (10 mg/kg, 24 hours DLI, 100 J/cm^2^, 100 mW/cm^2^). Scale bars = 500 µm. **E,** Quantification of histopathologic photodamage 72 hours post-treatment of Cal-33 tumor xenograft using apoptosis marker cleaved caspase-3. Units %. Mean ± SD. *N* = 3–4 tumors/treatment group. Additional statistical analysis is provided in Supplementary Table S8. H&E, hematoxylin and eosin (stain for tissue microanatomy and cellular morphology); CD31, cluster of differentiation 31 (marker for vascular/endothelial cell); PIMO, pimonidazole (fluorescent marker for tissue hypoxia); DAPI, 4′,6-diamidino-2-phenylindole (nuclear DNA fluorescent marker); Pyro (fluorescent marker for PS nanoparticles); ^64^Cu tissue autoradiography (intensity marker for ^64^Cu-labeled PS concentration in tissues); %Cells Marker^+^ (percent of nucleated cells positive for marker of interest in tumor cross-section); TUNEL (DNA fragmentation marker).

To understand the mechanisms of PS-PDT damage and optimize treatment conditions, the pathophysiologic differences between the Cal-33 xenograft and syngeneic MOC22 tumor models and their impact on intratumoral distribution of PS were investigated. Brightfield images of frozen sections of Cal-33 and MOC22 tumors demonstrated very heterogeneous tissue architecture between the two model types (Fig. 4A): Cal-33 cell line tumors were characterized by islands of malignant cells surrounded by thick extracellular matrix in lesions, whereas syngeneic MOC22 tumors had significant cellular density (hypercellularity) and fine stroma. CD31 staining for endothelial cells (i.e., blood vessels) revealed sparse and scattered vascular supply in the Cal-33 tumors whereas in MOC22 tumors there was rich CD31 staining particularly in the dense hypercellular regions, although the perfusion of these vessels could not be inferred. Tissue hypoxia visualized with pimonidazole (PIMO) staining showed numerous hypoxic microregions distributed throughout the Cal-33 tumor, but only moderate and diffuse hypoxic regions in the MOC22 (Fig. 4A; Supplementary Fig. S4).

For histologic evaluation of intratumoral PS accumulation, high-resolution fluorescence microscopy imaging of PS fluorescence and tissue autoradiography for ^64^Cu-labeled PS radioactivity were used. From the results illustrated in Fig. 4B, the pattern of PS accumulation appeared greatest near the tumor periphery and deficient in the centers of both tumors. Another feature in the intratumoral accumulation of PS was their close spatial proximity to CD31-stained tumor blood vessels. In Fig. 4C, we evaluated the penetration distance of PS nanoparticles into tumor cells by measuring the shortest distance between fluorescent Pyro^+^ cell (positive for PS uptake) and CD31^+^ vascular endothelial cell. The resulting proximity distribution of CD31←Pyro distances were positively skewed toward the vessels with median distances of 37.8 µm and 27.8 µm in the Cal-33 and MOC22 tumors, respectively (Fig. 4C; Supplementary Fig. S5A). This observation is unsurprising given the known diffusional restrictions of 100-nm nanoparticles like PS through the dense extracellular collagen matrix of tumors (29).

Evaluating the intratumoral distribution of hypoxic PIMO^+^ cells [i.e., pO_2_ ≤10 mmHg (30)] in a similar proximity-based manner revealed an almost uniform distribution of PIMO^+^ cells with increasing distances away from CD31^+^ blood vessel (CD31←PIMO distribution in Fig. 4C; Supplementary Fig. S5B). This finding was counterintuitive because the threshold for diffusion restricted hypoxia in tissues is typically assumed > 70 µm from blood vessels (31). However, a limitation of our quantitative analysis was that the perfusion/functionality of blood vessels could not inferred from CD31^+^ staining alone, without correcting for which reportedly yields distributions of intratumoral hypoxia staining (32, 33) similar to Fig. 4C. Finally, measuring the distances between hypoxic PIMO^+^ cells and the nearest fluorescent Pyro^+^ cells (Pyro←PIMO distribution in Supplementary Fig. S5C) confirms the visual absence of significant overlaps in tumor macro/microregions of tissue hypoxia and PS uptake in Fig. 4A and B. Altogether the histologic analysis of PS distribution supports a mechanism of damage from PS-PDT localized to normoxic cell populations close to the tumor vasculature.

Finally, confirmation of the photosensitization of tumors following PS administration was performed in the subcutaneous Cal-33 xenograft tumor model using histologic readouts of tissue damage after PS-PDT. Mice were treated with PS-PDT using surface illumination with 671-nm light and the tumors excised 3 days later for histopathologic analysis. From brightfield images of the treated tumors in Fig. 4D, there was obvious evidence of extensive tissue damage from changes in tumor microanatomy on H&E, and confirmation with staining for cellular apoptosis using TUNEL. Quantifying the extent of cellular damage using quantitative pathologic image analysis similarly confirmed significant apoptosis with cleaved caspase-3 staining in 65.7% ± 17.3% of tumor cells treated with PS-PDT versus only 3.9% ± 5.0% and 8.9% ± 3.7% apoptotic tumor cells in the light and drug treatment tumors, respectively (Fig. 4E, *P* value <0.001; see statistics in Supplementary Table S8).

### PS-PDT Efficacy in Tumor-bearing Mouse Models of Oral Cavity Cancer

The efficacy of PS nanoparticle-mediated PDT treatments was established in subcutaneous mouse tumors treated with a single PDT treatment using surface illumination at 671-nm wavelength through the overlying skin. Clinical responses and changes in tumor volume were monitored for 14 days post-treatment. Four treatment groups were randomized from 53 Cal-33 tumors: 20 PS-PDT, 17 untreated controls, nine PS drug controls, and seven light controls. In the MOC22, two treatment groups were randomized from 24 animals: 11 PS-PDT, and 13 untreated controls.

**FIGURE 5:**
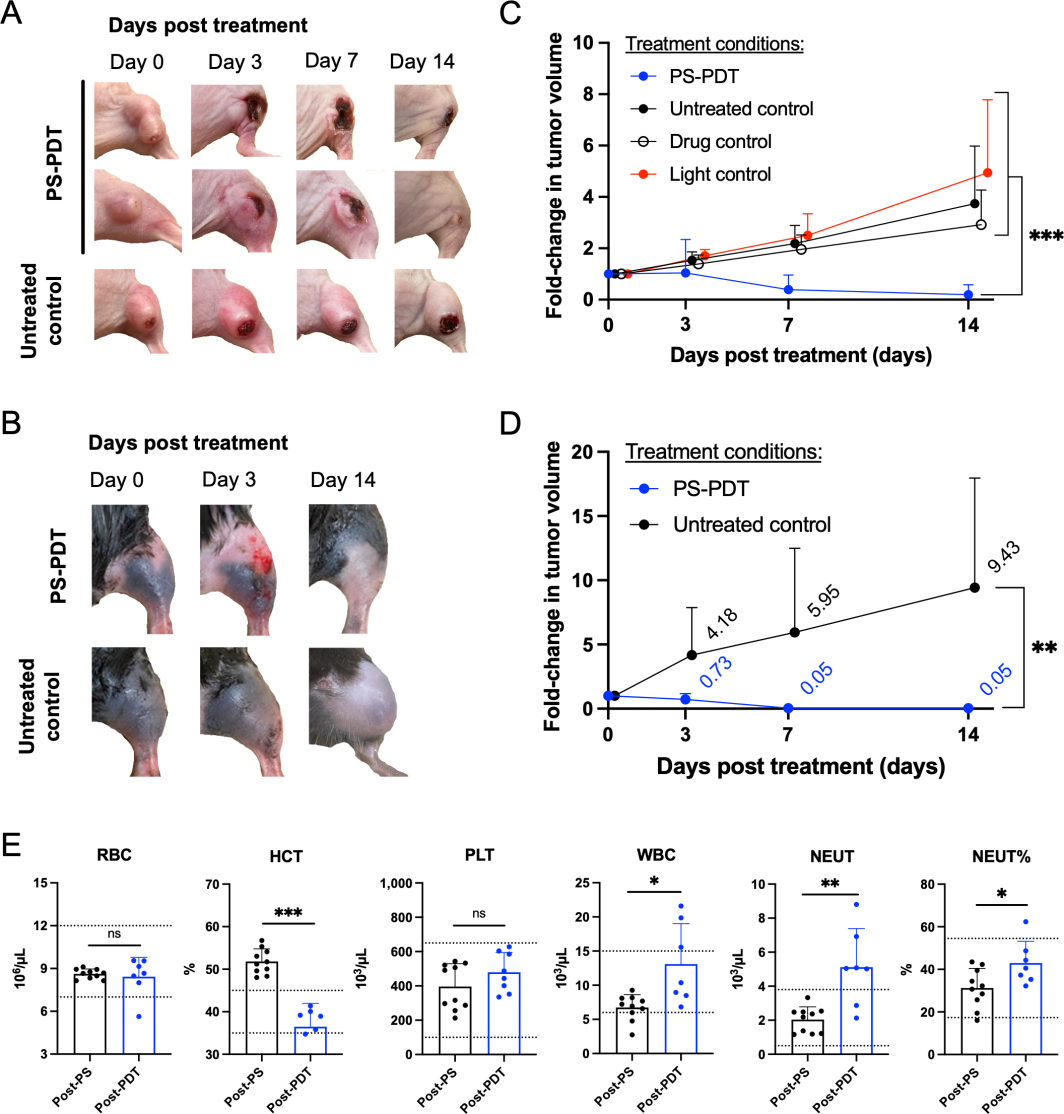
Antitumor treatment response to PS-PDT (10 mg/kg, 24 hour DLI, 100 J/cm^2^) in tumor-bearing mice models of oral cavity cancer. Representative posttreatment images in subcutaneous Cal-33 tumors (**A**) and in subcutaneous MOC22 tumors (**B**) following treatment on day 0. **C,** Fold change in tumor volume in subcutaneous Cal-33 tumors following treatment on day 0. *N* = 20 tumors (PS-PDT), 17 tumors (untreated control), nine tumors (drug control), and seven tumors (light control). **D,** Fold change in tumor volume in subcutaneous MOC22 tumors following treatment on day 0. *N* = 11 tumors (PS-PDT), and 13 tumors (untreated control). For C and D, Unitless. Mean + SD. Day 14 statistics: multiple unpaired (two-sample) *t* tests using Holm-Šídák correction method for multiple comparisons and ⍺ = 0.05. Additional PS-PDT efficacy data and statistical analysis are provided in Supplementary Tables S9 and S10. **E,** Hematologic changes in immune competent MOC22 tumor-bearing mice 24 hours post-PS administration (“Post-PS”) and 72 hours post-PS-PDT treatment (“Post-PDT”). Units given. Bar plot with mean + SD. ● represent individual replicates. Normal limits for each parameter represented by dotted lines. *N* = 10 Post-PS mice and 8 Post-PDT mice. Statistics: unpaired *t* tests without correction for multiple comparisons and ⍺ = 0.05. Additional hematology and blood chemistry analysis is provided in Supplementary Fig. S7. RBC, red blood cells (× 10^6^ cells/µL); HCT, hematocrit (%); PLT, platelets (× 10^3^/µL); WBC, white blood cells (× 10^3^ cells/µL); NEUT, neutrophil count (× 10^3^ cells/µL); NEUT%, relative neutrophil count (%).

Representative images of Cal-33 (Fig. 5A) and MOC22 tumors (Fig. 5B) revealed a potent antitumor response in PS-PDT treated animals compared with the untreated ones. On day 3 after PS-PDT, the targeted treated areas depicted marked edema, skin erythema, and evidence of tumor necrosis. By day 7, the hindlimb swelling had resolved, and eschar covered the treated area (Fig. 5A), and on day 14 the area healed almost *ad-integrum*. In the untreated control tumors, there was unrestricted tumor growth that by day 14 created challenges for animal ambulation (Fig. 5A and B). There was no evidence of cutaneous photosensitivity (e.g., skin erythema, swelling of paws and ears) from exposure to conventional room lighting observed in any mice administered PS.

Significant reductions in the volumes of Cal-33 and MOC22 tumor were observed in PS-PDT treated animals compared with control animals. On day 14 post-treatment, the volume of Cal-33 tumors in the PS-PDT treated group had reduced to 30.1 ± 84.5 mm^3^ or 20% ± 38% their original volume on day 0 whereas the untreated control group had grown to 343.4 ± 200.4 mm^3^ or 3.74 ± 2.24-fold (Fig. 5C). The residual disease in the PS-PDT treated tumors 14 days post-treatment were 73.4% ± 7.7% viable cells on histopathologic analysis (Supplementary Table S8), which hints that these partially responding tumors could be a risk of recurrence beyond the 14 day follow-up period completed here. The volumes of Cal-33 tumors in the drug and light control groups had similarly increased on average by 2.92 ± 1.35 and 4.95 ± 2.84-fold, respectively. The reduction in Cal-33 tumor volume in the PS-PDT treated group was statistically significant on day 14 compared with the experimental controls (*P* value < 0.001, see statistics in Supplementary Table S9).

In MOC22 tumors, by day 14 post-treatment tumor volumes in the PS-PDT treated group had reduced to 3.9 ± 12.8 mm^3^ (5 ± 15%) their original volume whereas the untreated control group had expanded to 212.5 ± 168.2 mm^3^ or 3.74 ± 2.24-fold (Fig. 5D). The reduction in MOC22 tumor volume on day 14 compared with the untreated control was statistically significant (*P* value = 0.004, see statistics in Supplementary Table S10).

Complete tumor responses were obtained in 13/20 (65%) and 10/11 (91%) of PS-PDT treated Cal-33 and MOC22 tumors respectively by day 14 post-treatment (Supplementary Fig. S6). Partial tumor responses were achieved in 5/20 (25%) and 1/11 (9%) of PS-PDT treated Cal-33 and MOC22 tumor, respectively. Only two PS-PDT treatment failures were observed in a Cal-33 tumor-bearing mice: 1/20 (5%) no response and 1/20 (5%) progressive disease in the PS-PDT treated group. None (0%) of the Cal-33 tumors in the untreated, drug, and light control groups achieved complete responses by day 14 post-treatment. Notably, apparent complete tumor responses were obtained in 2/13 (15%) of untreated MOC22 tumors (see Supplementary Fig. S6B), which is consistent with literature, reported variability with establishing this syngeneic murine tumor model (34).

To investigate the safety of PS-PDT, clinical pathology was performed with blood and plasma samples collected from immune competent MOC22 tumor-bearing mice after PS nanoparticle administration and 3 days after PS-PDT treatment (as in Fig. 5E). No changes in red blood cell and platelet levels were detected at either timepoint, whereas a clinically meaningful drop in hematocrit—likely a consequence of hypervolemia related to post-PDT swelling and inflammation at the tumor site—and trending increases in circulating white blood cells and neutrophils were observed 3 days after PS-PDT treatment (Fig. 5E). These were consistent with expected hematologic changes following PDT in mice (35). Values for other hematologic and clinical chemistry parameters remained stable and within physiologic normal levels (Supplementary Fig. S7).

### PS-PDT Efficacy in an Orthotopic Rabbit Model of Oral Cavity Cancer

**FIGURE 6:**
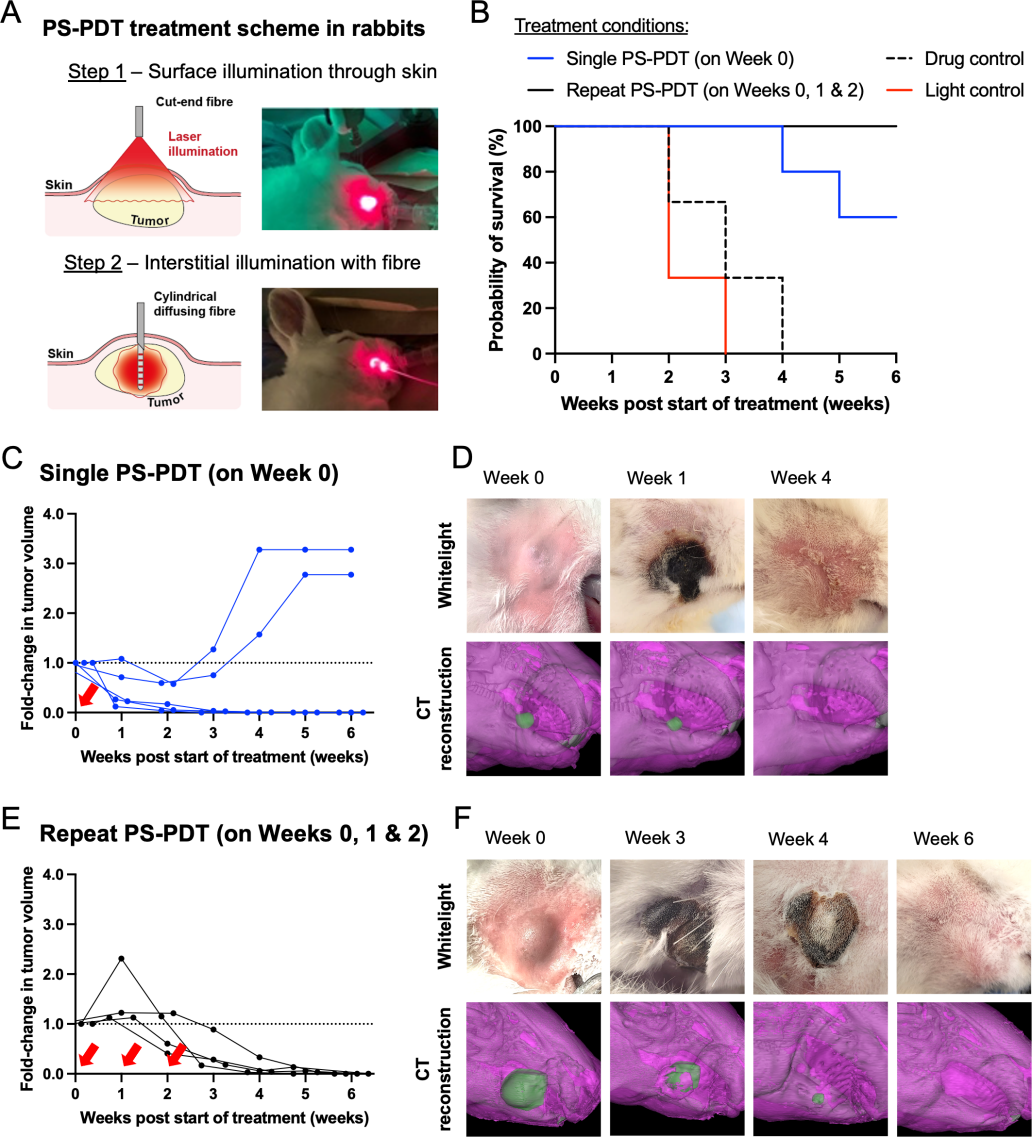
Antitumor treatment response to PS-PDT (10 mg/kg, 24 hour DLI, 200 J total) in an orthotopic VX-2 rabbit tumor model. **A,** Overview of “two-step” PDT treatment scheme in rabbits involving first, surface illumination of the tumor through the overlying skin using an external laser beam (100 J/cm^2^, 100 mW/cm^2^); and second, interstitial illumination with a diffusing fiber (1 cm, 100 J/cm, 100 mW/cm) inserted into the center of the tumor mass. **B,** Survival analysis of buccal VX-2 tumors following start of treatment on week 0. Event was tumor volume >3.05 cm^3^ (humane endpoint). *N* = 5 tumors (single PS-PDT), 4 tumors (repeat PS-PDT), 3 tumors (drug control), and 3 tumors (light control). Statistics: multiple comparisons of survival curves performed with log-rank tests using Bonferroni correction method for multiple comparison and ⍺ = 0.05. Single PS-PDT versus repeat PS-PDT = ns; Single PS-PDT versus drug control = ns; Single PS-PDT versus light control = *; Repeat PS-PDT versus drug control = *; Repeat PS-PDT versus light control = **. Fold change in tumor volume (**C**) and representative white light images and CT image reconstructions (**D**) of orthotopic buccal VX-2 tumors following single PS-PDT treatment on week 0. Fold change in tumor volume (**E**) and representative white light images and CT image reconstructions (**F**) following repeat PS-PDT treatments on weeks 0, 1, and 2. For C and E, red arrows indicated occasions of PS-PDT treatment. For D and F, normal anatomy outlined in purple, VX-2 tumor highlighted in green. Images not scaled for size. Additional PS-PDT efficacy data and statistical analysis are provided in Supplementary Table S11.

To demonstrate the efficacy of PS nanoparticle–mediated PDT treatments in a larger scale, higher fidelity animal model of oral cavity cancer, a proof-of-concept experiment of PS-PDT was performed in an orthotopic VX-2 rabbit tumor model. Fifteen male white rabbits bearing VX-2 tumors in the buccinator muscle were randomized into four treatment groups: 5 rabbits undergoing a single PS-PDT treatment on week 0 (labeled “single PS-PDT”), 4 rabbits undergoing three repeat weekly PS-PDT treatments for the same tumor on weeks 0, 1, and 2 (labeled “repeat PS-PDT”), 3 drug controls, and 3 light controls. The volumes of the VX-2 tumors at the time of randomization were 639 ± 349 mm^3^ (Supplementary Table S11), or about 5–10x larger than the subcutaneous mouse tumors treated in Fig. 5. Given the limited penetration of 671-nm laser light in tissues of about 7 mm, to adequately treat these deeply seated approximately 10-mm-wide lesions we implemented a “two-step” PDT treatment scheme combining surface and interstitial PDT as outlined in Fig. 6A.

The antitumor responses to single and repeat PS-PDT treatments in the orthotopic VX-2 tumor rabbit model are demonstrated in Fig. 6B–F. In the single PS-PDT treatment group, the treated buccal area exhibited obvious tumor necrosis and eschar formation 1 week post-treatment and by week 2 all rabbits exhibited partial tumor responses with an average reduction in tumor volume of 71 ± 28% (Fig. 6C). Over the next 4 weeks, the buccal tumors in 3/5 (60%) rabbits achieved completely ablation with no radiological evidence of locoregional recurrence on weeks 4, 5, and 6. The eschar in the treated area naturally soughed off and unveiled skin with good cosmetic appearance and minor evidence of scaring (Fig. 6D).

However, in 2/5 (40%) rabbits in the single PS-PDT group, tumors began to progress after week 2 and eventually reached volumes > 3.05 cm^3^ (humane endpoint) on weeks 4 and 5 (Fig. 6C), which was still longer than the median survival observed in the drug control (3 weeks) and light control (2 weeks) groups (see Supplementary Fig. S8). Two explanations are offered for this observed divergence in responses to single PS-PDT treatment: first, the initial tumor volumes of the 2 rabbits that failed were significantly larger than the 3 who achieved complete response (1,174 ± 110 mm^3^ vs. 736 ± 143 mm^3^, *P* value = 0.037). Second, the rapid growth of the orthotopic VX-2 model is known to result in a necrotic tumor core that is unresponsive light-based ablative techniques (24) and which may have been present in the two rabbit tumors who failed single PS-PDT treatment.

To overcome the potential variability in treatment responses related to underlying heterogeneity in the orthotopic tumors, we implemented a repeat PS-PDT treatment plan administering the same treatment parameters [i.e., PS dose, drug-light interval (DLI), light dose] in the same tumors for 3 consecutive weeks. The average starting tumor volume in the repeat PS-PDT group was 1,088 ± 555 mm^3^ (Supplementary Table S11). After the first treatment, the clinical responses were suboptimal with tumor volumes increasing on average by 1.45 ± 0.58-fold to 1,443 ± 558 mm^3^ (Fig. 6E). After the repeated PS-PDT treatments on weeks 1 and 2, the tumor volumes in all rabbits began to gradually decrease and a large eschar formed. Finally on week 6, all 4/4 (100%) of the VX-2 tumors were completely ablated and the treated buccal area appeared fully healed with minimal scaring (Fig. 6F).

Altogether these efficacy data of single and repeat PS-PDT treatments in a large scale orthoptic rabbit model demonstrate the safety and tolerability of three repeat weekly PS-PDT treatments to successfully eliminate deeply seated 10 mm lesions in the oral cavity with clinically satisfactory functional and cosmetic outcomes.

## Discussion

Our group has previously reported the development of PS, a phototheranostic nanoparticle assembled from a multifunctional building block consisting of chlorin photosensitiser–conjugated phospholipids (a.k.a., porphyrin-lipid conjugates; ref. 17). Porphyrin-lipid conjugate is a isometrically pure synthetic compound that is biodegradable *in vivo* (36) and possess a strong absorption at 671-nm wavelength (ε_670_ ∼45,000 L/mol·cm) with penetration depths of up to 7 mm in tissues (Fig. 2C). Unique to PS nanoparticles are their structurally driven photophysical and photochemical properties (Fig. 1B): disassembly of the nanoparticle, typically following uptake in cells, results in a 100-fold increase in fluorescence intensity at 675 nm and an approximately 4-fold increase in cytotoxic singlet oxygen generation (Fig. 2D and E). The unique ability for PS fluorescence imaging and PDT reactivity to be activated in pathologic tissues imparts additional selectivity to PS-mediated treatments compared with other molecular photosensitizers like mTHPC and porfimer sodium that are “always on” from the moment of administration.

In this study, three multispecies models of oral cavity cancer were selected to evaluate the pharmacokinetics and photosensitization of intravenously administered PS nanoparticles. Each tumor model possessed a distinctive macroanatomy and microanatomy, pathophysiology and immunology which allowed us to assess safety and antitumor efficacy of PS-PDT treatments in different contexts. Unsurprisingly, the dissimilarities in tumor characteristics gave rise to differences in the PS pharmacokinetic profiles and tumor selectivity (Fig. 3). Absolute PS concentrations in syngeneic MOC22 tumors was 4-fold greater than in Cal-33 xenograft tumors 24 hours post-injection, and highest in the orthotopic VX-2 rabbit tumors despite equivalent mg/kg doses of PS in all three models (Supplementary Fig. S2). Differences in nanoparticle accumulation between human xenograft and syngeneic allograft tumor models have been inconsistently reported in literature (37) and which of these preclinical tumors best predicts the uptake of PS in OCSCC lesions in patients remains to be seen.

PS nanoparticles exhibited superior selectivity for tumor tissues 24 hours post-injection compared with other tissues types associated with the oral cavity, including skin, muscle, fat, and nerves (brain; Fig. 3E and F). An alternative expression of photosensitizer selectivity is tumor-to-skin concentration ratio, which were 1.22, 8.86, and 4.06 for the Cal-33, MOC22, and VX-2 tumor models, respectively. For comparison, the tumor-to-skin concentration ratios for mTHPC (0.15 mg/kg, i.v.) ranged from 1.79 to 4.81 in feline models of spontaneous OCSCC (38). PS fluorescence from *ex vivo* tissues similarly confirmed efficient and tumor selective activation of PS nanoparticles 24 hours post-injection (Fig. 3G and H) and provides motivating evidence for the use of intraoperative PS fluorescence imaging to plan and guide PS-PDT treatments in patients with OCSCC (39, 40).

Following systemic administration, PS predominantly accumulated along the periphery or rim of subcutaneous Cal-33 and MOC22 tumors (Fig. 4B). A similar pattern of intratumoral PS distribution has also been observed previously in VX-2 rabbit tumors (39). Porfimer sodium reportedly exhibited a similar “edge-weighted” pattern after intravenous administration in an SCCVII squamous cell carcinoma model (41). Approximately 3.8% and 9.3% of nucleated cells in the cross-sections of Cal-33 and MOC22 tumors were positive for PS nanoparticle fluorescence, respectively, and the majority of these cells were located next to blood vessels (Fig. 4C). The identity of the cell types taking up PS was not determined in this study but was likely a combination of cancer cells and tumor-associated macrophages (42, 43). The close proximity of PS nanoparticles to tumor blood vessels is consistent with observations for physicochemically similar nanoparticles (44, 45) and implies the transport of PS into the tumor interstitium was primarily diffusion limited.

The Cal-33 xenograft tumors used for optimizing PS-PDT treatment parameters exhibited patterns of hypoxic microregions (Fig. 4A) similar to those observed in OCSCC patient biopsies (46). Approximately 8.8% of all nucleated cells in the Cal-33 tumor were hypoxic (Fig. 4C), which suggests this tumor type should be responsive to oxygen-dependent PDT treatments. Indeed, subcutaneous Cal-33 xenograft tumors were effectively photosensitized 24 hours after PS injection and PS-PDT resulted in significant apoptosis in 65.7% of tumor cells 72 hours post-treatment (Fig. 4E).

The exact mechanisms of PS-mediated PDT photodamage could not be conclusively determined in this study design. The extensive apoptotic staining with TUNEL throughout the PS-PDT treated tumors (Fig. 4D) contrasted with the intraumoral distribution of PS along the periphery (Fig. 4B; Supplementary Fig. S4) suggests a combination of mechanisms including direct tumor cell kill and vascular damage to tumor endothelial cells consistent with the transport pathways of nanoparticles into tumor tissues (20). Inflammatory reactions after PS-PDT demonstrated by edema and eschar formation in the treated area (Fig. 5A and B) and changes in blood leukocytes and neutrophils in PS-PDT treated immune competent MOC22 tumor-bearing mice (Fig. 5E) provide evidence for post-treatment immune reactions that warrants further investigation (47, 48).

High antitumor efficacy of a single PS-PDT was reproducibly demonstrated in all three tumor models of oral cavity cancer. Complete tumor responses (complete eradication) following PS-PDT treatment were achieved in 65% and 91% of Cal-33 and MOC22 tumors, respectively, at 14 days post-treatment (Fig. 5C and D). No tumor recurrences were detected in these animals during follow-up, although we note that a 2-week period may be insufficient to detect recurrences in these models (49). These efficacy results are superior to PDT treatments in human squamous cell xenograft models reported using porfimer sodium—which demonstrated significant treatment lethality in mice and a 43%–60% complete response rate (49, 50)—and mTHPC, which exhibited a 43%–100% tumor recurrence rates 8 to 24 days post-PDT and adverse side-effects in mice following drug administration and PDT treatment (49, 51). No fatalities were observed in any animals following PS-PDT and changes in hematology and blood chemistry after PS administration and PS-PDT treatment were either within normal physiologic levels or consistent with literature reported changes post-PDT in mice (Supplementary Fig. S7).

In the orthotopic VX-2 rabbit tumor model, a treatment scheme combining surface and interstitial PDT achieved complete tumor ablation in 60% (3/5) of rabbits after a single treatment and disease progression in 40% of rabbits after 3 weeks post-PDT (Fig. 6B and C). Repeating PS-PDT treatments for the same tumor weekly for 3 weeks were safe and well tolerated by rabbits and improved complete response rates to 100% (4/4) of treated buccal tumors (Fig. 6E). Functional and cosmetic outcomes in single and repeat PS-PDT treated rabbits were also clinically acceptable and confirm the suitably of PS-PDT for sparing healthy mucosal tissues in the oral cavity (Fig. 6D and F). Administration of PS in healthy male rabbits with 3-fold higher dose than the 10 mg/kg phototherapeutic dose used for PS-PDT treatments did not prompt any long-lasting changes in hematology, hepatic function, or blood biochemistry values outside normal levels (Supplementary Fig. S9) nor result in any clinically significant tissue findings 30 days after administration (Supplementary Table S12).

The antitumor efficacy results of single and repeat PS-PDT in VX-2 rabbit models are similar to reported outcomes with mTHPC and PEGylated liposomal mTHPC (FOSPEG) in felines with spontaneous OCSCC (38, 52), a veterinary model of OCSCC with great translational relevance to human disease that our group plans to explore in the future. Our group has previously reported curative treatments in orthoptic VX-2 rabbit tumors using PS nanoparticle–mediated photothermal ablation (40): a nonsurgical treatment modality that heats tissues to above the thermal coagulation of proteins (>55°C) resulting in necrotic cell death. Although thermal ablation is a promising treatment modality for certain solid cancers, in the context of the oral cavity the selectivity of PDT treatments provides clinically superior therapeutic, functional, and cosmetic advantages for OCSCC versus nonspecific thermal therapies (53, 54).

In this study, we demonstrated the safety and therapeutic efficacy of PS nanoparticle–mediated PDT treatments in three preclinical models of OCSCC with distinctive microanatomy and pathophysiology. PS nanoparticles reproducibly exhibited selective uptake and structurally driven fluorescence and PDT activation in tumor tissues and cells. OCSCC tumors were effectively photosensitized 24 hours after PS injection, resulting in significant cellular apoptosis throughout the tumor following PDT illumination. A remarkable 90% (36/40) overall response rate from PS-PDT treatments was reported across all three tumor models without local recurrences after achieving complete clinical response, nor evidence of any drug or treatment related adverse effects during the follow-up period. Taken altogether, the safety and tumor selectivity of PS-PDT treatments confirmed in multiple OCSCC models combined with the NIR fluorescence of PS for image-guided treatment planning are persuasive for their continued clinical translation as a tissue sparing ablation modality in early-stage OCSCC.

## Supplementary Material

Supplementary Methods All supplementary methods.

Supplementary Tables All supplementary tables.

Figure S1 Supplementary figure 1 and legend.

Figure S2 Supplementary figure 2 and legend.

Figure S3 Supplementary figure 3.

Figure S4 Supplementary figure 4 and legend.

Figure S5 Supplementary figure 5 and legend.

Figure S6 Supplementary figure 6 and legend.

Figure S7 Supplementary figure 7 and legend.

Figure S8 Supplementary figure 8 and legend.

Figure S9 Supplementary figure 9 and legend.
