# Supplementary material for: Nanoparticle-mediated Photodynamic Therapy as a Method to Ablate Oral Cavity Squamous Cell Carcinoma in Preclinical Models
Source: Cancer Res Commun. 2024 Mar 15;4(3):796–810. doi: 10.1158/2767-9764.CRC-23-0269 (PMC10941731; doi:10.1158/2767-9764.CRC-23-0269)
Supplement: Supplementary Methods — All supplementary methods. [file crc-23-0269-s01.pdf]

TITLE: Nanoparticle mediated photodynamic therapy as a method to ablate oral cavity squamous cell carcinoma in preclinical models

AUTHOR LIST: Axel Sahovaler[1,2,3,†], Michael S. Valic[3,4,†], Jason L. Townson[2,3], Harley H.L. Chan[2,3], Mark Zheng[3], Sharon Tzelnick[1,2,3], Tiziana Mondello[1,2,3], Alon Pener-Tessler[1,2,3], Donovan Eu[1,2,3], Abdullah El-Sayes[3], Lili Ding[3], Juan Chen[3], Catriona M. Douglas[1,2,5], Robert Weersink[2,3,6], Nidal Muhanna[1,2,7], Gang Zheng[3,4,6,*], Jonathan C. Irish[1,2,3,*]

[1]Department of Otolaryngology–Head and Neck Surgery, University of Toronto, Toronto, ON, Canada.
[2]TECHNA Institute, Guided Therapeutics (GTx) Program, University Health Network, Toronto, ON, Canada.
[3]Princess Margaret Cancer Centre, University Health Network, Toronto, ON, Canada.
[4]Institute of Biomedical Engineering (BME), University of Toronto, Toronto, ON, Canada.
[5]Department of Otolaryngology–Head and Neck Surgery, Queen Elizabeth University Hospital, Glasgow, United Kingdom.
[6]Department of Medical Biophysics, University of Toronto, Toronto, ON, Canada.
[7]Department of Otolaryngology–Head and Neck Surgery, Tel Aviv Sourasky Medical Centre, Tel Aviv University, Tel Aviv, Israel.
[†]Authors contributed equally to work.

[*]Corresponding authors: Gang Zheng, Princess Margaret Cancer Centre, University Health Network, 101 College Street, TMDT RM 5-354, Toronto, ON M5G 1L7, Canada. Phone: 416-581-7666; Fax: 416-581-7667. E-mail: gang.zheng@uhnres.utoronto.ca; Jonathan C. Irish, University of Toronto, 190 Elizabeth Street, Toronto, ON M5G 2C4, Canada. Phone: 416-340-3113. E-mail: jonathan.irish@uhn.ca.

RUNNING TITLE: Nanoparticle-mediated PDT for OSCC ablation

KEYWORDS: nanoparticles; photodynamic therapy; precision medicine; oral cavity squamous cell carcinoma;

PS nanoparticles

PORPHYSOME [PEGylated porphyrin-lipid conjugate containing nanoparticles] (PS) nanoparticles (NanoMedicine Fabrication Center) were supplied as a sterile ready-to-use product suspended in 1x phosphate buffered saline with pH 7.2. 64Cu-PORPHYSOMES (^{64}Cu-PS) nanoparticles were prepared by radiolabelling the pre-formed PS nanoparticles with positron-emitting Copper-64 (^{64}Cu) metal in a "one-pot" reaction: $^{64}Cu(II)Cl_2$ (Washington University School of Medicine) supplied in 0.1 M HCl solution was diluted with 0.2 M sodium acetate (Sigma-Aldrich 32319), pH 5.5 solution before being added to PS nanoparticles with volume ratio 1:1. The specific activity of ^{64}Cu-PS nanoparticle at the time of labelling was ~ 49.7 ± 13.9 ×10^9 Bq/mol (on basis of mols of porphyrin-lipid conjugate). The mixture was heated to ~55 °C for 60 minutes and then chilled on ice after labelling. The labelling efficiency of ^{64}Cu-PS was confirmed using instant thin layer chromatography (iTLC): replicate aliquots of ^{64}Cu-PS were dilute 1:4 in 0.05 M ethylenediaminetetraacetic acid (EDTA) (Sigma-Aldrich E9884) and then separated on heat activated silica gel plates (Agilent SGI0001) using 0.05 M EDTA as the chromatography solvent. The PS-bound ^{64}Cu remains at the origin (R_f ^{64}Cu-PS ~0.14) whereas the unbound/free ^{64}Cu metal migrate with the solvent front (R_f free ^{64}Cu > 0.88). After development, the iTLC plate was cut in two and the top (free ^{64}Cu) and bottom (^{64}Cu-PS) sections measured with a gamma counter (PerkinElmer Wallac WIZARD 3" 1480). The ratio of ^{64}Cu radioactivity between top and bottom sections was used to calculate the labelling efficiency. The acceptance criteria for ^{64}Cu-PS was labelling efficiency not less than 90% (grand mean of triplicate samples). ^{64}Cu-PS nanoparticles were used without further purification. PS and ^{64}Cu-PS were diluted in 0.9% sodium chloride injection (Baxter 1301) prior to administration to animals.

Physicochemical characterisation

The physicochemical characteristics of PS and ^{64}Cu-PS nanoparticles are detailed in **Supplementary Table 1**. Measurement of ^{64}Cu-PS samples were performed following storage at 2–8 °C until the sample radioactivity had completely decayed (e.g., ~10x half-lives of ^{64}Cu or ~6 days). The morphology PS and ^{64}Cu-PS nanoparticles were visualised with transmission electron microscopy (TEM) (FEI Tecnai) on formvar coated grids (Electron Microscopy Sciences FCF400-Cu-UB) after negative staining with 2.0 w/v% uranyl acetate (Electron Microscopy Sciences 224002). The lipid composition of PS and ^{64}Cu-PS were determined with ultra-performance liquid chromatography–evaporative light scatting (UPLC-ELS, Waters ACQUITY): replicate aliquots of PS and ^{64}Cu-PS were diluted in methanol (Sigma-Aldrich 154903) and passed through a BEH C18 reverse-phase chromatography column (Waters 186002350) running a mobile phase consisting of an acidic ammonium acetate (Supelco AX1222) and methanol mixture. The molecular weights of PS and ^{64}Cu-PS nanoparticles were measured in 1x phosphate buffered saline (PBS) (Corning 21-031-CV) carrier buffer by centrifugal field-flow fractionation–multi-angle light

scatting/dynamic light scattering (cFFF-MALS/DL) (Postnova CF2000): the retention times of replicate nanoparticle samples were analysed using a constant field method varying the field strength between 1373 g (2,250 rpm) and 2,692 g (4,500 rpm). The average effective molar mass (M_{50}) was derived assuming a spherical particle model with a ~5-nm thick bilayer (from TEM measurements) and an aqueous-filled core. pH measurements were performed on PS and ^{64}Cu-PS samples without dilution and using a micro pH electrode (Thermo Scientific 9810BN). The hydrodynamic diameter and polydispersity index (PDI) of PS and ^{64}Cu-PS dilute in distilled water were measured in a glass cuvette (Malvern Panalytical PCS1115) using dynamic light scattering (DLS) with a 532-nm wavelength 'green' laser (Malvern Panalytical Zetasizer Nano). The zeta (ζ)-potential of PS and ^{64}Cu-PS dilute in distilled water were measured in a dip cell (Malvern Panalytical ZEN1002) using phase analysis light scattering (PALS) with a 532-nm wavelength 'green' laser.

PS and ^{64}Cu-PS nanoparticle physiological stability experiments

The stabilities of PS and ^{64}Cu-PS nanoparticles were evaluated under simulated physiological conditions: particle samples were dilute in 50 v/v% foetal bovine serum (FBS) (Gibco 12483020) (or in 1x PBS as a control) and maintained at 37 °C with constant shaking. For size stability analysis, PS samples at each incubation timepoint were filtered through a dextran-agarose Superdex 200 gel column (GE Health Care Life Sciences 17-1043-02) to remove unbound serum proteins (i.e., high molecular weight PS nanoparticles have shorter elution times than low molecular weight serum proteins). PS samples were collected from the column and the sample PDI measured using DLS. For ^{64}Cu chelation stability analysis, ^{64}Cu-PS samples at each incubation timepoint were dilute 1:4 in 0.05 M EDTA and the free ^{64}Cu separated from PS-bound ^{64}Cu using iTLC assay for labelling efficiency described previously. An experimental control for possible serum protein-bound ^{64}Cu was included at each timepoint. The radiochemical purity of ^{64}Cu-PS samples (corrected as needed for any protein-bound ^{64}Cu) was measured and reported at each timepoint as percent change in purity normalised to the initial sample purity at 0 h. Regression analysis of data from 1 h to 48 h was performed using a one phase decay model and plateau = 0 % (i.e., complete ^{64}Cu-PS dissociation at time infinity) to estimate the dissociation kinetics of ^{64}Cu from ^{64}Cu-PS nanoparticles in PBS or 50 v/v% FBS.

Photophysical and photochemical characterisation

The absorbance spectra of PS and ^{64}Cu-PS nanoparticles were measured using UV-Vis spectrophotometry (Agilent Cary 60), and the fluorescence spectra measured using fluorescence spectrometry (Horiba FluoroMax) with excitation wavelength 416-nm. The spectra of 'intact' PS and ^{64}Cu-PS nanoparticles were measured in distilled water, whereas the spectra of disassembled PS monomers (i.e.,

porphyrin-lipid conjugate) were measured in aqueous medium containing 1.0 v/v% non-ionic surfactant (Sigma-Aldrich X100). Measurement of ^{64}Cu-PS samples were performed following storage at 2–8 °C until the sample radioactivity had completely decayed (e.g., ~10x half-lives of ^{64}Cu or ~6 days). The molar attenuation (ε) profile of intact and disassembled PS was derived from linear regression of the absorption spectra of serial diluted PS in aqueous media from 0.1–200 μM and reported on the basis of mols of porphyrin-lipid conjugate.

The structurally dependent singlet oxygen ($^1O_2$) generation of PS nanoparticles was measured using singlet oxygen sensor green (SOSG) assay (Thermo Fisher S36002). SOSG reagent was freshly prepared in methanol (100 mM) and mixed with PS samples (1~2 μM porphyrin-lipid conjugate amount) before dilution in either 1x PBS (intact nanoparticle) or 1.0 v/v/% non-ionic surfactant (Sigma-Aldrich X100) to yield a final SOSG concentration of 10 μM. Replicate samples were aliquoted onto a 96-well black polystyrene microplate (Corning 3915) and illuminated with a 671-nm diode pumped solid state (DPSS) laser (LaserGlow Technologies R6710B1FX) using fluences ranging from 0.5 J/cm^2 to 10 J/cm^2 (50 mW/cm^2 rate constant). Negative control wells containing SOSG only in 1x PBS or 1.0 v/v/% non-ionic surfactant were included. Following laser illumination, the fluorescence of unquenched SOSG was quantified with a plate reader (BMG Labtech CLARIOstar Plus) using 488-nm excitation wavelength and 525-nm emission wavelength. Note there is no PS fluorescence within this emission window (see spectra in **Figure 2D**). Fluorescence readings were corrected for negative controls and the statistics from five independent experiments reported.

Whole blood and plasma pharmacokinetics

In tumour-bearing mice, the blood clearance of PS (10 mg/kg, 400-500 MBq ^{64}Cu/kg, IV) was assessed in cohorts of 5 mice/model using serial sampling from the saphenous vein at 9~10 timepoints post-injection. At each timepoint, approximately ~0.025 mL of whole blood was drawn from the vein with a heparinised capillary tube (Fisherbrand 41B22362566). The ^{64}Cu radioactivity in 0.010 mL aliquots of whole blood and of centrifuge-separated plasma were assayed using a gamma counter (PerkinElmer Wallac WIZARD 3" 1480). The activity of ^{64}Cu was decay-corrected to the time of injection and concentration of PS reported as percent injected dose per mL (%I.D./mL) or as μg/mL after multiplying by the injected dose of porphyrin-lipid conjugate. ^{64}Cu is a surrogate measure for PS concentration in vivo owing to the stable ^{64}Cu-PS chelation under physiological conditions (see **Supplementary Figure 1G**).

The plasma pharmacokinetics of PS (10 mg/kg, IV) was assessed in healthy and VX-2 tumour-bearing rabbits (6x rabbits total) using serial sampling from the marginal ear vein at 8~10 timepoints post-infusion. At each timepoint, approximately ~0.500 mL of whole blood was drawn from the vein with a heparinised capillary tube (Sarstedt 20.1345.100) and the plasma separated with centrifugation. Replicate

samples of plasma were diluted in aqueous buffer containing 1.0 v/v% non-ionic surfactant (Sigma-Aldrich X100) and the fluorescence intensity of PS measured at 675-nm emission wavelength (see spectra in **Figure 2D**) using fluorescence spectrometry (Horiba FluoroMax). Fluorescence intensity was converted to porphyrin-lipid conjugate concentration using a standard curve with ~0.01 μg/mL detection limit and corrected for sample dilution. The plasma concentration of PS was normalised to the injected dose of porphyrin-lipid conjugate and reported as μg/mL or as %I.D./mL after normalising to the injected dose of porphyrin-lipid conjugate.

Pharmacokinetic parameters in **Figure 3A,B**, **Supplementary Table 2** and **Supplementary Table 3** were calculated with noncompartmental analysis in MatLab (The MathWorks Version 9.13.0.2105380) using a standard two stage approach. Descriptive statistics (mean ± standard deviation) were reported.

Tissue distribution profile

In tumour-bearing mice, the tissue biodistribution of PS (10 mg/kg, 400-500 MBq ^{64}Cu/kg, IV) was assessed in cohorts of 5~10 mice/model 24 hours post-injection. Mice were euthanised with cardiac puncture and cervical dislocation technique under inhaled anaesthesia. Gross necropsy was performed on each animal and representative samples of major organs and the subcutaneous tumour were collected. Collected tissues were weighted and measured for ^{64}Cu radioactivity using an automated gamma counter (PerkinElmer Wallac WIZARD 3" 1480) with a linear detection limit for ^{64}Cu of ~800 Bq using 60 sec integration time. Activity measurements were radioactive decay-corrected to the time of PS nanoparticle injection and expressed as a percentage of the injected dose per gram of tissue (%I.D./g) or as μg/g after multiplying by the injected dose of porphyrin-lipid conjugate. ^{64}Cu is a surrogate measure for PS concentration in vivo owing to the stable ^{64}Cu-PS chelation under physiological conditions (see **Supplementary Figure 1G**).

The tissue biodistribution of PS (10 mg/kg, IV) was assessed in 3x VX-2 tumour-bearing rabbits 24 hours post-infusion. Rabbits were euthanised with pentobarbital sodium administration (100 mg/kg, IV) under inhaled anaesthesia. Gross necropsy was performed on each animal and representative samples of major organs and the orthotopic tumour were collected. Replicate samples of each tissue were weighed (~0.1 g each) and homogenised with a tissue homogeniser (Bertin Technologies Precellys Evolution) using 2.0-mm zirconia beads (BioSpec 11079124ZX). The tissues were homogenised in an aqueous-based tissue solubiliser (PerkinElmer 6NE9100) and were kept at room temperature overnight following homogenisation to extract the porphyrin-lipid conjugate. Afterwards, the samples were centrifuged to separate the beads and tissue debris, and the clear tissue homogenate collected, diluted in 1x PBS and measured with fluorescence spectrometry (Horiba FluoroMax). The fluorescence intensity of PS measured at 675-nm was converted to homogenate PS concentration using a three-point standard addition method

and 2 µg/mL standard 'spike' solution of porphyrin-lipid conjugate in dimethylsulfoxide (Sigma-Aldrich D8418). The tissue concentration of PS was back calculated from the dilution-corrected homogenate concentration and the known tissue weight, and averaged across replicate tissue samples. Lastly, PS tissue concentration was normalised to the injected dose of porphyrin-lipid conjugate and reported as µg/g or as %I.D./g after normalising to the injected dose of porphyrin-lipid conjugate.

Ex vivo tissue fluorescence

The fluorescence signal of PS (10 mg/kg, IV) in select organs and tumour tissues was imaged *ex vivo* 24 hours post-nanoparticle administration. Animals were feed a regular diet containing alfalfa prior to imaging. Freshly excised tissues and tumours were rinsed to remove excess blood and placed on black matted paper. Muscle tissue was always included in the field of view of fluorescence images for image analysis (described below). For mice tissues, the surface fluorescence of *ex vivo* tissues were measured with a multispectral imaging system (Xenogen IVIS Imaging System 200 Series) using 675-nm excitation and 720-nm emission wavelength channels, and 1.00 second exposure time. PS tissue fluorescence was captured on both surfaces of each tissue (e.g., "top" and "reverse" sides) and the signal analysed using regions of interest over each tissue to calculate the average radiant efficiency with procedurally defined units: (photons/sec/cm^2/str)/(µW/cm^2). Tissue autofluorescence was subtracted using an automated program and 580–610-nm background filters. The radiant efficiencies from both surfaces of each tissue were averaged and normalised to the signal in the muscle, and the tissue-to-muscle fluorescence ratio reported. For rabbit tissues, the surface fluorescence of *ex vivo* tissues were imaged using an *in vivo* endoscopic fluorescence imaging system (Novadaq PINPOINT) customised with a 675-nm excitation and 720-nm long pass emission filter. An instrument reported intensity value ranging from 0–300 was measured on both surfaces of each tissue, and the average PS tissue fluorescence normalised to the signal in the muscle. The tissue-to-muscle tissue fluorescence ratio was reported.

Histologic evaluation of tumour microanatomy

Mice with subcutaneous Cal-33 xenograft and syngenetic MOC22 tumours approximately ~100 mm^3 in volume were euthanised with carbon dioxide. The tumours and healthy tissues were excised, embedded in optimal cutting temperature compound (Fisher HealthCare 4585) and flash frozen in isopropanol cooled by liquid nitrogen. The frozen tissue blocks were serially sectioned with slice thickness of 4 µm using a cryostat and transferred onto glass microscope slides. Staining with haematoxylin and eosin (H&E) was performed as per laboratory standard operating procedures. Immunohistochemistry staining with horseradish peroxidase conjugated anti-CD31 (Novus NBP1-71663H) was performed according to the

suppliers' protocol and visualised using DAB substrate (Novus NBP3-18633). Whole slide brightfield scans (Leica Biosystems Aperio AT2) were acquired with 20x resolution.

Pimonidazole hydrochloride (PIMO) (Hypoxyprobe HP) was prepared as a non-sterile solution in 0.9% sodium chloride injection (Baxter 1301) with concentration 100 mg/mL and stored at 2–8°C protected from light until use. Tumour-bearing mice with tumours approximately ~100 mm^3 in volume were administered PIMO (60 mg/kg, IV) via tail vein injection ~60 minutes prior to euthanasia with carbon dioxide. Excised subcutaneous Cal-33 and MOC22 tumours and healthy tissues were embedded in OCT compound and flash frozen in isopropanol cooled by liquid nitrogen. The frozen tissue blocks were serially sectioned with slice thickness of 4 μm using a cryostat and transferred onto glass microscope slides. Staining with 4′,6-diamidino-2-phenylindole (DAPI) (Invitrogen R37606) was performed according to the suppliers' protocol. Immunohistochemistry staining for PIMO was performed with rabbit polyclonal IgG1 (Hypoxyprobe HP PAb2627) and counter stained with Alexa Flour 555 conjugated anti-rabbit IgG (Invitrogen A-21428) according to the suppliers' protocol. Whole slide fluorescence scans (Zeiss Axioscan 7) using DAPI and Cy3 filters were acquired with 20x resolution.

Histologic evaluation and quantitative analysis of intratumoural PS distribution

Mice with subcutaneous Cal-33 xenograft and syngenetic MOC22 tumours approximately ~100 mm^3 in volume were administered PS (10 mg/kg, 400-500 MBq ^{64}Cu/kg, IV) via tail vein injection and euthanised 24 hours post-injection with carbon dioxide. The subcutaneous tumours and healthy tissues were excised, embedded in OCT compound and flash frozen in isopropanol cooled by liquid nitrogen. The frozen tissue blocks were serially sectioned with slice thickness of 4 μm using a cryostat and transferred onto glass microscope slides. Staining with DAPI was performed according to the suppliers' protocol. Whole slide fluorescence scans (Zeiss Axioscan 7) using DAPI and Cy5 filters were acquired with 20x resolution; note the Cy5 filter (Ex 620-nm, Em 700-nm, BPF 660-nm) was suitable for the fluorescence spectrum of PS nanoparticles (labelled "Pyro", see spectra in **Figure 2D**). Tissue autoradiography for ^{64}Cu-labelled PS radioactivity was performed by mounting tissue slides into an exposure cassette with a high-resolution storage phosphor screen (PerkinElmer 7001487). Screens were developed overnight and imaged afterwards with 600 DPI (~42 μm/pixel) resolution using a phosphor imaging system (PerkinElmer Cyclone Plus). Note that ^{64}Cu is a surrogate measure for PS nanoparticle concentration in vivo since ^{64}Cu remains stably chelated by porphyrin-lipid conjugate under physiological conditions (see **Supplementary Figure 1G).**

For quantitative analysis of PS intratumoural distribution, digitised brightfield and fluorescent histology slides with CD31 (chromogenic), PIMO and PS (labelled "Pyro") fluorescent markers were analysed using spatial proximity analysis (Indica Labs HALO). Tissue histology slides containing one or two chromogenic or fluorescent markers on the same slide were first co-registered to one another using a

combination of algorithmic approaches and manual adjustment using visual landmarks (e.g., tissue boundaries, blood vessel, etc.). Second, analysis of co-registered slides was performed using CytoNuclear FL algorithm to identify nucleated cells positive for the desired marker (only one marker per cell). The percentage of nucleated cells in the tumour cross-section positive for a desired marker was calculated and reported as mean ± standard deviation (see **Figure 4C**). Results obtained from CytoNuclear FL analysis were inputted into the spatial analysis algorithm evaluating the 'proximity' between different markers on separate co-registered histology slides. The spatial relationships for markers Pyro to CD31 (CD31–Pyro), PIMO to CD31 (CD31←PIMO), Pyro to PIMO (PIMO←Pyro) and PIMO to Pyro (Pyro←PIMO) were analysed (see **Supplementary Figure 5**). Output of the spatial analysis were individual distances in µm for each cell positive for the second marker in the direction of the nearest positive cell for the first marker: for example, analysis of CD31←Pyro spatial relationship yields the shortest distances between $Pyro^+$ cell (2nd marker) to the nearest $CD31^+$ cell (1st marker). Note that a positive cell with the 1st marker could serve as a point of reference for multiple cells positive with the 2nd marker. The maximum distance cut-off for the analysis was 100 µm. Analysis outputs from tumours of the same type were naïvely pooled together and descriptive statistics (mean, median, quartiles, skewness) for each tumour type reported.

Histologic evaluation and quantitative analysis of PS-PDT photodamage

Subcutaneous Cal-33 xenograft tumour-bearing mice with tumour volumes approximately ~100 mm^3 were administered PS (10 mg/kg, IV) via tail vein injection followed by PDT treatment 24 hours post-injection. The tumours were externally irradiated using a 671-nm DPSS laser (LaserGlow Technologies R6710B1FX) and a cut-end fibre with a 9-mm diameter for a fluence of 100 J/cm^2 (100 mW/cm^2 fluence rate). Mice receiving no treatment/intervention (untreated control), receiving PS administration only (drug control), or surface PDT laser illumination only (light control) were also included as treatment groups. Three days following PS-PDT or control treatments, the animals were euthanised with carbon dioxide and the tumours excised for histopathological analysis for photodamage. Partially responding tumours to PS-PDT treatment after fourteen days were also analysed. Tumour tissues were fixed in formalin solution (Sigma-Aldrich HT501128) for three days before transferred into alcohol solution (Sigma-Aldrich R8382) and paraffin embedded. Tissue blocks were serially sectioned with slice thickness of 4 µm and transferred onto glass microscope slides. Staining H&E was performed as per laboratory standard operating procedures. Terminal deoxynucleotidyl transferase dUTP nick end labelling (TUNEL) was performed according to the suppliers' protocol (Roche 03333566001, Roche 11093070910) and was visualised using DAB substrate (Abcam ab64238). Immunohistochemistry staining with anti-cleaved caspase-3 (Cell Signaling Technology 9661) was performed according to the suppliers' protocol and counterstained with biotinylated anti-rabbit IgG (Vector Laboratories BA-1000-1.5) and DAB substrate according to the suppliers' protocols. Whole

slide brightfield scans of H&E, TUNEL, and cleaved caspase-3 stained tumour sections were acquired with 20x resolution.

Quantification of cleaved caspase-3 staining in tumour histology from the PS-PDT treated group (Day 3 and Day 14) and untreated, drug, and light control groups was performed using histological image analysis software (Indica Labs HALO). A cellular classifier was created using a random forest machine learning algorithm to classify different types of tissues/cells within the tumour microenvironment based on the image colour and texture. The tissue/compartments classified included whitespace, necrosis, tumour, and healthy tissues based on provided examples. Analysis output consisted of the areas of the different tissue types, total number of cells, and the percentage of cleaved caspase-3 positive cells within each tissue (see **Supplementary Table 8**). The percentage (%) of classifier-identified tumour cells positive for cleaved caspase-3 staining was calculated, and the mean ± standard deviation for the PS-PDT treated (Day 3 and Day 14), and untreated, drug, and light control groups (N=2~3 tumours analysed/treatment group) were reported.

1. Safety assessments of PS-PDT in treated MOC22 tumour-bearing mice

The haematology and clinical chemistry were evaluated in immunocompetent C57BL/6 WT mice with subcutaneous MOC22 tumours at (i) 24 hours post-PS administration (10 mg/kg, IV), and at (ii) 72 hours after surface PS-PDT treatment (24 hour DLI, 100 J/cm^2, 100 mW/cm^2). Large volume blood samples were collected at each endpoint in a terminal procedure using lithium heparin collection tubes (Sarstedt 20.1209.100) for clinical chemistry and K3 ETDA collection tubes (Sarstedt 20.1278.100) for haematology. The heparinised blood samples were centrifuged, and the separated plasma analysed for comprehensive diagnostic profile using a biochemistry analyser (Abaxis VETSCAN VS2 Chemistry Analyzer). The EDTA whole blood sample was analysed for full blood count using a haematology analyser (Abaxis VETSCAN HM5 Haematology Analyzer). Normal limits for each clinical parameter in female C57BL/6 WT mice was obtained from The Jackson Laboratory (1).

Safety assessments of PS in healthy rabbits

The clinical and anatomical toxicology of single intravenous PS administration (30 mg/kg, IV) was evaluated in healthy male New Zealand white rabbits (Charles River). PS nanoparticles (~25 mL) were infused via the marginal ear vein over 10 minutes. Blood and plasma samples were collected from the contralateral ear vein pre-infusion (baseline) and 48 hours, 1 week and 4 weeks post-PS infusion. Plasma samples for toxicokinetic analysis were collected at these and additional timepoints at 5 minutes, 1 hour, 6 hours, and 24 hours post-infusion. Plasma samples were analysed for comprehensive diagnostic profile using a biochemistry analyser (Abaxis VETSCAN VS2 Chemistry Analyzer). The whole blood samples

were analysed for full blood count using a haematology analyser (Abaxis VETSCAN HM5 Haematology Analyzer). Normal limits for each clinical parameter in New Zealand white rabbits was obtained from Zoetis (2,3). 4 weeks post-PS administration, final blood samples were collected and rabbits euthanised with pentobarbital sodium administration (100 mg/kg, IV) under inhaled anaesthesia. A gross necropsy was performed, and organs collected for anatomical pathology, including liver, kidney, spleen, heart, lungs, large and small intestines, brain, and eyeballs (globes). Tissues were formalin fixed for 7 days, paraffin embedded and H&E-stained slides prepared for histopathological analysis by a board-certified veterinarian. Slides from 5x PS nanoparticle treated rabbits were submitted for unblinded evaluation to a board-certified veterinary pathologist (see reporting in **Supplementary Table 12**). Changes in organs were graded on a scale from: 0 (none), 1 (minimal), 2 (mild), 3 (moderate), 4 (marked), and 5 (severe).

SUPPLEMENTARY METHODS REFERENCES

1. The Jackson Laboratory. Physiological Data Summary – C57BL/6J (000664) [Internet]. Available from: http://jackson.jax.org/rs/444-BUH-304/images/physiological_data_000664.pdf
2. Zoetis Services. VETSCAN® VS2 Reference Ranges (SI Units) [Internet]. 2021. Report No.: VTS-00038B R2. Available from: https://www.zoetisus.com/content/_assets/docs/Diagnostics/technical-papers/VETSCAN-VS2-Reference-Ranges-VTS-00038.pdf
3. Zoetis Services. VETSCAN® HM5 Reference Ranges (Additional Species) [Internet]. 2021. Report No.: VTS-00426. Available from: https://www.zoetisus.com/content/_assets/docs/Diagnostics/technical-papers/HM5-Reference-Ranges-iPad-VTS-00426.pdf
