# Supplementary material for: Nanoparticle-mediated Photodynamic Therapy as a Method to Ablate Oral Cavity Squamous Cell Carcinoma in Preclinical Models
Source: Cancer Res Commun. 2024 Mar 15;4(3):796–810. doi: 10.1158/2767-9764.CRC-23-0269 (PMC10941731; doi:10.1158/2767-9764.CRC-23-0269)
Supplement: Supplementary Tables — All supplementary tables. [file crc-23-0269-s02.pdf]

<u>TITLE</u>: Nanoparticle mediated photodynamic therapy as a method to ablate oral cavity squamous cell carcinoma in preclinical models

<u>AUTHOR LIST</u>: Axel Sahovaler[1,2,3,†], Michael S. Valic[3,4,†], Jason L. Townson[2,3], Harley H.L. Chan[2,3], Mark Zheng[3], Sharon Tzelnick[1,2,3], Tiziana Mondello[1,2,3], Alon Pener-Tessler[1,2,3], Donovan Eu[1,2,3], Abdullah El-Sayes[3], Lili Ding[3], Juan Chen[3], Catriona M. Douglas[1,2,5], Robert Weersink[2,3,6], Nidal Muhanna[1,2,7], Gang Zheng[3,4,6,*], Jonathan C. Irish[1,2,3,*]

[1]Department of Otolaryngology–Head and Neck Surgery, University of Toronto, Toronto, ON, Canada.
[2]TECHNA Institute, Guided Therapeutics (GTx) Program, University Health Network, Toronto, ON, Canada.
[3]Princess Margaret Cancer Centre, University Health Network, Toronto, ON, Canada.
[4]Institute of Biomedical Engineering (BME), University of Toronto, Toronto, ON, Canada.
[5]Department of Otolaryngology–Head and Neck Surgery, Queen Elizabeth University Hospital, Glasgow, United Kingdom.
[6]Department of Medical Biophysics, University of Toronto, Toronto, ON, Canada.
[7]Department of Otolaryngology–Head and Neck Surgery, Tel Aviv Sourasky Medical Centre, Tel Aviv University, Tel Aviv, Israel.
[†]Authors contributed equally to work.

[*]Corresponding authors: Gang Zheng, Princess Margaret Cancer Centre, University Health Network, 101 College Street, TMDT RM 5-354, Toronto, ON M5G 1L7, Canada. Phone: 416-581-7666; Fax: 416-581-7667. E-mail: gang.zheng@uhnres.utoronto.ca; Jonathan C. Irish, University of Toronto, 190 Elizabeth Street, Toronto, ON M5G 2C4, Canada. Phone: 416-340-3113. E-mail: jonathan.irish@uhn.ca.

<u>RUNNING TITLE:</u> Nanoparticle-mediated PDT for OSCC ablation

<u>KEYWORDS:</u> nanoparticles; photodynamic therapy; precision medicine; oral cavity squamous cell carcinoma;

Supplementary Table 1. Physicochemical characterisation of PS and ^{64}Cu-labelled PS nanoparticles.

**Parameter**	**Instrument**	**Units**	**PS nanoparticles, day 0 stability (N=3 samples)**		**PS nanoparticles, 18-month stability (N=3 samples)**		**^{64}Cu-labelled PS nanoparticles† (N=2 samples)**	
			**Mean**	**S.D.**	**Mean**	**S.D.**	**Mean**	**S.D.**
Porphyrin-lipid conjugate mass ratio	UPLC-ELS	%	68.43	2.54	65.16	8.62	65.28	10.86
Cholesterol mass ratio	UPLC-ELS	%	16.96	0.68	18.75	2.21	17.97	2.99
MPEG-2000-DPSE mass ratio	UPLC-ELS	%	14.61	0.45	16.09	1.21	16.75	2.90
Molecular weight	cFFF-MALS/DLS	MDa	N/D	N/D	146.0	9.9	145.0	10.3
pH	pH meter	-	7.10	0.05	7.01	0.20	6.33	0.07
Diameter	DLS	nm	98.99	0.39	101.82	0.39	104.49	5.17
Polydispersity index	DLS	-	0.115	0.010	0.147	0.009	0.160	0.035
Zeta potential	PALS	mV	-25.4	2.0	-22.3	1.1	-22.4	3.4

$^{\dagger 64}$Cu-PS samples stored at 2–8 °C until radioactivity had completely decayed (e.g., ~5x half-lives of ^{64}Cu) before measurement.

Abbreviations: cFFF-MALS/DLS, Centrifugal field flow fractionation-Multi-angle light scattering/Dynamic light scattering; DLS, Dynamic light scatting; UPLC-ELS, Ultra-performance liquid chromatography-Evaporative light scatting detector; MPEG-2000-DPSE, 1,2-Distearyol-*rac*-glycerol-3-Phosphoethanolamine-N-Polyethyleneglycol-2000; N/D, Not done; PALS, Phase-analysis light scattering.

Supplementary Table 2. Plasma pharmacokinetics of PS (IV bolus or short infusion) in subcutaneous Cal-33 xenograft and syngeneic MOC22 tumour mouse models, and in orthotopic VX-2 tumour-bearing and healthy New Zealand white rabbits. Parameters calculated using noncompartmental analysis.

**Model type**		**Cal-33 tumour-bearing mice**		**MOC22 tumour-bearing mice**		**VX-2 tumour-bearing rabbits**		**Healthy rabbits**	
**Dose (mg/kg)**		10		10		10		30†	
**Reference weight (kg)**		0.020		0.020		3.50		3.50	
**N**		5		5		6		4	
**Parameter**	**Units**	**Mean**	**S.E.**	**Mean**	**S.E.**	**Mean**	**S.E.**	**Mean**	**S.E.**
λ_z	h^{-1}	0.071	0.004	0.052	0.008	0.028	0.001	0.019	0.001
Adjusted R^2		0.926	0.030	0.981	0.007	0.901	0.054	0.999	0.010
AUC_{last}	%I.D./mL·h	648	31	734	20	16.5	0.5	58.6	7.9
C_{max}	%I.D./mL	75.4	5.3	93.6	6.7	0.772	0.037	0.907	0.030
MRT	h	12.5	0.6	13.3	0.6	39.71	0.55	50.61	1.92
$T_{1/2}$	h	9.9	0.6	14.3	2.2	27.54	0.40	37.23	1.07
AUC_{∞}	%I.D./mL·h	673	33	765	17	25.55	1.28	58.59	7.91
CL	mL/h	0.150	0.007	0.131	0.003	4.09	0.17	1.71	0.18
	mL/h·kg	7.50	0.35	6.55	0.15	1.17	0.05	0.49	0.05
V_z	mL	2.11	0.07	2.71	0.45	153.2	6.5	91.7	11.8
	mL/kg	106	3.50	136	22.5	43.8	1.86	26.2	3.37
V_{ss}	mL	1.87	0.10	1.74	0.11	153.1	5.8	86.4	6.6
C_0	%I.D./mL	81.5	10.4	97.6	7.1	0.776	0.037	0.913	0.031

†PS drug dose used for toxicity and toxicokinetic study in healthy rabbits.

Abbreviations: λ_z, terminal rate constant; AUC_{last}, Area under concentration-time curve from time = 0 to last time point; C_{max}, maximum observed concentration; MRT, Mean residence time; $T_{1/2}$, Terminal half-life; AUC_{∞}, Total area under the concentration-time curve extrapolating to t_{∞}; CL, Total drug clearance; V_z, Volume of distribution; V_{ss}, Apparent volume of distribution at equilibrium; C_0, Extrapolated concentration at time = 0.

Supplementary Table 3. PS (10 mg/kg, 400 MBq ^{64}Cu/kg, IV) tissue concentrations in subcutaneous Cal-33 xenograft tumour models 24 hours post-injection. Units N=5~10 mice/tissue.

Tissue	N	%I.D./g†			S.U.V.‡			μg/g$		
		Mean	S.D.	Max	Mean	S.D.	Max	Mean	S.D.	Max
Adrenals	5	4.029	2.685	8.739	0.899	0.548	1.853	10.51	7.008	22.81
Bile	4	18.26	9.027	31.34	4.176	2.064	7.114	47.65	23.56	81.80
Bladder wall	5	1.186	0.529	1.987	0.270	0.122	0.261	3.096	1.381	5.186
Blood	5	3.305	0.359	3.610	0.749	0.060	0.802	8.627	0.936	9.422
Bone	5	1.282	0.201	1.514	0.291	0.041	0.324	3.347	0.524	3.952
Brain	5	0.248	0.078	0.322	0.056	0.016	0.073	0.648	0.203	0.840
Fat	5	0.696	0.246	1.096	0.156	0.047	0.232	1.815	0.642	2.861
Heart wall	5	2.582	0.382	3.178	0.586	0.083	0.721	6.738	0.996	8.295
Intestines	5	3.307	0.661	4.018	0.755	0.174	0.956	8.631	1.726	10.49
Kidneys	5	5.464	0.641	6.380	1.243	0.158	1.448	14.26	1.674	16.65
Liver	5	21.60	2.259	24.61	4.897	0.364	5.217	56.37	5.897	64.23
Lungs	5	3.532	0.978	4.482	0.796	0.192	1.004	9.217	2.552	11.70
Lymph nodes	5	4.417	2.104	7.774	0.989	0.423	1.648	11.53	5.491	20.29
Muscle	10	0.521	0.052	0.889	0.119	0.040	0.210	1.292	0.392	2.107
Ovaries	5	1.429	0.738	2.298	0.3194	0.155	0.487	3.730	1.927	5.998
Pancreas	5	1.234	0.322	1.458	0.279	0.070	0.331	3.222	0.842	3.805
Plasma	5	3.840	1.351	4.898	0.864	0.282	1.075	10.02	3.525	12.78
Skin	5	2.103	0.615	3.117	0.475	0.121	0.661	5.488	1.606	8.135
Spleen	5	24.69	7.122	30.03	5.662	1.813	7.105	64.45	18.59	78.38
Stomach	5	3.119	0.702	4.050	0.710	0.168	0.919	8.140	1.832	10.57
Tumour	10	3.280	0.4361	5.738	0.751	0.331	1.303	8.074	3.110	13.60
Urine	5	0.605	0.287	1.107	0.138	0.069	0.261	1.579	0.749	2.889
Uteri	5	2.769	0.769	3.682	0.625	0.155	0.836	7.228	2.006	9.610

†%I.D. radioactive decay-corrected from time of assay to time of ^{64}Cu-PS administration.

‡Ratio of tissue ^{64}Cu-PS concentration and whole-body concentration of administered ^{64}Cu-PS (i.e., standardised uptake value).

$Calculated from %I.D./g tissue measurement and the known PS dose of porphyrin-lipid conjugate (mg).

Supplementary Table 4. PS (10 mg/kg, 500 MBq ^{64}Cu/kg, IV) tissue concentrations in subcutaneous syngeneic MOC22 tumour models 24 hours post-injection. Units N=5 mice/tissue.

Tissue	N	%I.D./g†			S.U.V.‡			µg/g$		
		Mean	S.D.	Max	Mean	S.D.	Max	Mean	S.D.	Max
Adrenals	5	4.863	1.292	6.608	1.073	0.319	1.500	11.52	3.061	15.66
Bile	5	30.51	16.94	51.97	6.663	3.708	11.28	72.31	40.14	123.2
Bladder wall	4	2.073	0.989	3.376	0.460	0.230	0.756	4.914	2.344	8.001
Blood	5	3.596	0.576	4.496	0.790	0.146	1.021	8.523	1.366	10.66
Bone	5	2.392	0.248	2.643	0.523	0.046	0.591	5.668	0.587	6.264
Brain	5	0.173	0.065	0.245	0.038	0.015	0.055	0.410	0.153	0.581
Fat	5	0.267	0.071	0.365	0.058	0.013	0.073	0.633	0.169	0.865
Heart wall	5	2.952	0.856	3.922	0.649	0.202	0.890	6.997	2.028	9.295
Intestines	5	4.430	0.583	5.267	0.971	0.139	1.196	10.50	1.383	12.48
Kidneys	5	7.678	2.388	10.06	1.693	0.569	2.283	18.23	5.670	23.88
Liver	5	21.04	1.565	23.25	4.608	0.364	5.208	49.87	3.709	55.10
Lungs	5	2.394	0.803	3.443	0.521	0.160	0.694	5.674	1.903	8.160
Lymph nodes	5	5.294	2.744	9.749	1.166	0.633	2.213	12.55	6.504	23.11
Muscle	5	0.339	0.104	0.508	0.073	0.019	0.102	0.802	0.246	1.204
Ovaries	5	1.721	0.349	2.236	0.378	0.083	0.501	4.078	0.827	5.299
Pancreas	5	0.745	0.272	1.074	0.165	0.065	0.241	1.766	0.641	2.545
Plasma	5	5.085	0.667	6.078	1.115	0.167	1.380	12.05	1.580	14.40
Skin	5	1.521	0.149	1.784	0.332	0.017	0.501	3.605	0.353	4.228
Spleen	5	25.11	7.753	36.95	5.542	1.885	8.387	59.51	18.37	87.57
Stomach	5	3.073	0.534	3.943	0.675	0.130	0.883	7.284	1.265	9.345
Tumour	5	13.67	5.742	20.51	2.977	1.182	4.198	32.40	13.61	48.61
Urine	4	0.505	0.332	0.830	0.112	0.073	0.180	1.197	0.787	1.967
Uteri	5	1.874	0.772	2.986	0.412	0.176	0.669	4.442	1.830	7.077

†%I.D. radioactive decay-corrected from time of assay to time of ^{64}Cu-PS administration.

‡Ratio of tissue ^{64}Cu-PS concentration and whole-body concentration of administered ^{64}Cu-PS (i.e., standardised uptake value).

$Calculated from %I.D./g tissue measurement and the known PS dose of porphyrin-lipid conjugate (mg).

Supplementary Table 5. PS (10 mg/kg, IV) tissue concentrations in orthotopic VX-2 rabbit tumour models 24 hours post-injection. N=3 rabbits/tissue.

Tissue	N	%I.D./g†			S.U.V.‡			µg/g		
		Mean	S.D.	Max	Mean	S.D.	Max	Mean	S.D.	Max
Adrenals	3	0.182	0.066	0.238	5.967	2.180	7.807	61.97	22.94	77.18
Aorta	3	0.096	0.034	0.071	3.135	1.110	4.400	32.01	20.76	43.48
Bile	3	0.292	0.174	0.521	9.557	5.656	17.10	41.47	35.77	95.07
Bladder wall	3	0.053	0.022	0.078	1.753	0.706	2.555	17.81	11.38	25.26
Bone marrow	3	0.038	0.003	0.040	1.246	0.107	1.317	12.35	2.458	13.02
Brain	3	0.027	0.005	0.031	0.874	0.149	1.030	8.499	3.048	10.18
Fat	3	0.064	0.011	0.073	2.102	0.368	2.402	20.78	10.96	23.74
Heart wall	3	0.045	0.011	0.056	1.463	0.354	1.825	14.48	6.270	18.04
Intestines	3	0.111	0.032	0.137	3.630	1.049	4.498	37.05	7.751	44.47
Kidneys	3	0.144	0.023	0.158	4.728	0.743	5.184	46.73	12.99	51.25
Liver	3	0.389	0.020	0.411	12.75	0.640	13.48	126.1	32.89	133.3
Lungs	3	0.245	0.084	0.331	8.036	2.753	10.86	79.59	31.48	107.4
Lymph nodes	3	0.070	0.003	0.072	2.295	0.099	2.347	22.76	7.626	23.30
Muscle	3	0.014	0.001	0.015	0.452	0.024	0.479	4.473	1.561	4.735
Pancreas	3	0.053	0.012	0.062	1.722	0.403	2.029	16.64	6.550	20.06
Plasma	3	0.347	0.053	0.431	10.59	1.613	13.16	121.6	18.5	151.0
Prostate	3	0.087	0.061	0.153	2.867	1.997	5.026	29.80	18.89	49.68
Salivary gland	3	0.064	0.003	0.068	2.095	0.108	2.216	20.75	3.673	21.91
Skin	3	0.058	0.011	0.070	1.899	0.364	2.296	18.77	7.949	22.70
Spleen	3	0.702	0.183	0.852	23.00	6.013	27.94	227.4	55.79	276.2
Stomach	3	0.087	0.028	0.116	2.837	0.917	3.819	28.05	13.11	37.67
Thyroid	3	0.094	0.045	0.142	3.097	1.464	4.643	30.18	12.25	45.89
Tumour	3	0.235	0.037	0.268	7.715	1.199	8.790	76.26	12.01	86.90
Urine	3	0.008	0.005	0.013	0.263	0.161	0.426	2.593	1.621	4.214

†Calculated from µg/g tissue measurement and the known PS dose of porphyrin-lipid conjugate (mg).

‡Ratio of tissue PS concentration and whole-body concentration of administered PS (i.e., standardised uptake value).

Supplementary Table 6. Tissue-to-muscle normalised concentration of PS (10 mg/kg, 400~500 MBq ^{64}Cu/kg, IV) in ex vivo tissues from subcutaneous Cal-33 xenograft and syngeneic MOC22 tumour mouse models, and of PS (10 mg/kg, IV) from orthotopic VX-2 rabbit tumour models 24 hours post-injection. Unitless. N=5~10 mice/tissue. N=3 rabbits/tissue.

Tissue	Cal-33 mouse model				MOC22 mouse model				VX-2 rabbit model			
	Mean	S.D.	Max	N	Mean	S.D.	Max	N	Mean	S.D.	Max	N
Adrenals	10.1	4.31	27.0	5	15.1	4.43	19.3	5	13.2	4.87	18.0	3
Aorta	-	-	-	-	-	-	-	-	6.93	2.48	10.2	3
Bile	40.7	6.32	59.5	4	102	77.0	216	5	21.1	12.6	39.4	3
Bladder wall	2.50	0.40	3.77	5	6.30	3.67	10.2	4	3.88	1.57	5.89	3
Blood	7.40	1.10	11.1	5	11.2	2.71	13.6	5	-	-	-	-
Bone	2.90	0.49	4.67	5	7.42	1.55	9.41	5	-	-	-	-
Bone marrow	-	-	-	-	-	-	-	-	2.75	0.28	3.04	3
Brain	0.55	0.12	0.98	5	0.57	0.29	0.84	5	1.93	0.34	2.38	3
Fat	1.63	0.46	3.38	5	0.82	0.23	1.04	5	4.65	0.85	5.54	3
Heart wall	5.78	0.84	8.24	5	9.12	3.15	13.4	5	3.23	0.80	4.21	3
Intestines	7.55	1.35	10.8	5	13.7	2.74	16.2	5	8.02	2.36	10.4	3
Kidneys	11.9	1.04	15.0	5	25.1	11.9	37.8	5	10.5	1.73	12.0	3
Liver	48.5	7.48	76.0	5	66.0	16.6	87.5	5	28.2	2.04	31.1	3
Lungs	7.81	1.50	13.7	5	7.26	2.36	11.4	5	17.8	6.16	25.1	3
Lymph nodes	10.3	3.45	24.0	5	16.7	9.28	28.4	5	5.07	0.34	5.44	3
Muscle	1.00	0.00	1.00	10	1.00	0.00	1.00	5	1.00	0.07	1.11	3
Ovaries	3.30	1.04	7.09	5	5.31	1.25	6.75	5	-	-	-	-
Pancreas	2.67	0.38	4.03	5	2.35	0.89	3.24	5	3.806	0.912	4.681	3
Plasma	8.42	1.82	15.1	5	15.9	3.82	20.2	5	25.2	4.05	32.6	3
Prostate	-	-	-	-	-	-	-	-	6.34	4.43	11.6	3
Salivary gland	-	-	-	-	-	-	-	-	4.63	0.34	5.11	3
Skin	4.93	1.27	9.62	5	4.72	0.99	5.98	5	4.20	0.83	5.30	3
Spleen	54.7	8.73	80.5	5	77.7	25.1	108	5	50.8	13.6	64.5	3
Stomach	6.87	0.86	8.97	5	9.78	3.21	12.9	5	6.27	2.05	8.79	3
Thyroid	-	-	-	-	-	-	-	-	6.85	3.26	10.7	3
Tumour	6.70	1.00	13.5	10	40.8	16.3	68.6	5	17.1	2.80	20.3	3
Urine	1.29	0.24	2.10	5	1.74	1.39	3.45	4	0.58	0.36	0.98	2
Uteri	6.27	1.28	10.8	5	5.81	2.65	9.01	5	-	-	-	-

Supplementary Table 7. Tissue-to-muscle normalised fluorescence intensity of PS (10 mg/kg, IV) in ex vivo tissues from subcutaneous Cal-33 xenograft and syngeneic MOC22 tumour mouse models, and orthotopic VX-2 rabbit tumour models 24 hours post-injection. Unitless. For mouse models: Ex: 675 nm, Em: 720 nm, 1 second exposure time. N=5 mice/tissue. For rabbit model: Ex: 675 nm, Em: 720 nm long pass. N=3 rabbits/tissue.

Tissue	Cal-33 mouse model				MOC22 mouse model				VX-2 rabbit model			
	Mean	S.D.	Max	N	Mean	S.D.	Max	N	Mean	S.D.	Max	N
Adrenal gland	-	-	-	-	-	-	-	-	6.32	0.07	9.38	3
Aorta	-	-	-	-	-	-	-	-	2.63	0.06	3.94	3
Bladder wall	-	-	-	-	-	-	-	-	3.12	0.46	6.88	3
Bone marrow	-	-	-	-	-	-	-	-	2.63	0.18	4.44	2
Brain	0.39	0.25	0.92	5	1.00	0.20	1.40	5	0.65	0.01	1.00	3
Fat	0.612	0.27	1.53	5	0.99	0.29	1.61	5	1.57	0.221	3.63	3
Heart wall	1.36	0.49	3.09	5	2.10	0.34	2.85	5	2.59	0.09	4.13	3
Intestines	-	-	-	-	-	-	-	-	5.89	0.23	9.25	3
Kidneys	3.89	1.50	10.1	5	7.50	1.40	10.4	5	5.97	0.47	10.3	3
Liver	10.1	3.40	21.0	5	16.9	2.47	21.5	5	13.2	0.12	19.1	3
Lungs	3.89	2.41	12.4	5	4.41	0.75	6.06	5	1.90	0.06	3.06	3
Lymph nodes	2.20	1.40	8.32	5	3.15	2.06	8.78	5	3.03	0.14	5.063	3
Muscle	1.00	0.43	1.00	5	1.00	0.18	1.00	5	1.00	0.07	1.00	3
Pancreas	1.90	0.69	4.39	5	2.74	0.93	4.74	5	4.74	0.47	9.38	3
Prostate	-	-	-	-	-	-	-	-	3.02	0.26	5.88	3
Salivary gland	-	-	-	-	-	-	-	-	2.91	0.20	5.38	3
Skin	4.29	1.50	9.16	5	2.77	0.67	4.24	5	0.46	0.02	0.75	3
Spleen	6.97	2.29	13.8	5	12.7	2.45	17.8	5	17.5	1.67	35.1	3
Stomach	-	-	-	-	-	-	-	-	3.38	0.74	7.69	3
Thyroid	-	-	-	-	-	-	-	-	1.88	0.14	3.19	2
Tumour	8.54	3.63	20.2	5	19.6	3.09	25.2	5	10.3	0.16	15.6	3

Supplementary Table 8. Quantitative histological evaluation of treatment response in subcutaneous Cal-33 xenograft tumours using cleaved caspase-3 (Casp3) staining.

Treatment condition (Day post-treatment)	Tissue area (mm^2)		Necrotic cells (%)		Viable cells (%)		Total viable cells ($x10^3$)		Cleaved $Casp3^+$ cells (%)		P value†
	**Mean**	**S.D.**	**Mean**	**S.D.**	**Mean**	**S.D.**	**Mean**	**S.D.**	**Mean**	**S.D.**	
Control	52.5	27.3	23.7	4.0	76.3	4.0	323.7	110.0	3.2	3.2	-
PS-PDT (Day 3)	32.7	19.0	33.9	4.4	66.1	4.4	144.6	69.9	65.7	17.3	<0.001
PS-PDT (Day 14)	10.6	15.1	26.6	7.7	73.4	7.7	89.9	108.9	0.4	0.3	0.99
Drug control	13.7	9.1	15.2	3.0	84.8	3.0	485.1	626.0	8.9	3.7	0.92
Light control	22.6	8.9	9.6	9.3	90.4	9.3	154.7	116.0	3.9	5.0	0.99

Description of treatment conditions:

Control – Untreated tumours

PS-PDT (Day 3) – Tumours resected 3 days following PS-PDT (10 mg/kg, 24-hour DLI, 100 J/cm^2, 100 mW/cm^2)

PS-PDT (Day 14) – Partially responding tumours resected 14 days following PS-PDT (10 mg/kg, 24-hour DLI, 100 J/cm^2, 100 mW/cm^2)

Drug control – Tumours resected 3 days following PS administration (10 mg/kg) only

Light control – Tumours resected 3 days following surface PDT light treatment (100 J/cm^2, 100 mW/cm^2) only

†Adjusted P value of comparison between mean percent cleaved $Casp3^+$ cells in Control group versus other treatment groups. Multiple unpaired (two-sample) t-tests using Holm-Šídák method correction for multiple comparisons and $\alpha = 0.05$. '-' denotes comparison not performed.

Supplementary Table 9. Antitumour treatment responses in subcutaneous Cal-33 xenograft tumour models following treatment on Day 0.

**Data type**			*Tumour volume (mm^3)*				*Fold-change in tumour volume*			
**Treatment condition**	**Days post treatment**	**N**	**Mean**	**Median**	**S.D.**	**P value†**	**Mean**	**Median**	**S.D.**	**P value†**
PS-PDT	0	20	108.1	89.8	62.5	-	1.00	1.00	0.00	-
	3	20	120.7	119.2	89.1	-	1.04	0.72	1.30	-
	7	20	34.9	0.0	47.2	-	0.39	0.00	0.57	-
	14	20	30.1	0.0	84.5	-	0.20	0.00	0.38	-
Untreated control	0	17	99.1	94.2	37.6	0.61	1.00	1.00	0.00	-
	3	17	153.1	148.5	71.2	0.46	1.54	1.46	0.33	0.14
	7	17	216.7	214.2	113.1	<0.001	2.19	2.08	0.71	<0.001
	14	17	343.4	365.6	200.4	<0.001	3.74	2.72	2.24	<0.001
Drug control	0	9	95.2	82.9	27.6	0.81	1.00	1.00	0.00	-
	3	9	130.9	130.1	40.0	0.81	1.39	1.38	0.35	0.43
	7	9	189.7	172.8	79.4	<0.001	1.96	2.07	0.56	<0.001
	14	9	265.9	259.6	113.6	<0.001	2.92	2.51	1.35	<0.001
Light control	0	7	96.9	100.1	47.9	0.67	1.00	1.00	0.00	-
	3	7	169.4	172.7	95.6	0.45	1.71	1.70	0.24	0.19
	7	7	242.0	215.0	148.8	<0.001	2.50	2.53	0.84	<0.001
	14	7	434.1	394.9	265.7	<0.001	4.95	3.94	2.84	<0.001

Description of treatment conditions:

PS-PDT – Tumour treated with PS-PDT (10 mg/kg, 24 hour DLI, 100 J/cm^2, 100 mW/cm^2) on Day 0

Untreated control – Untreated tumour

Drug control – Mouse administered PS only (10 mg/kg) on Day 0

Light control – Mouse administered surface PDT light treatment only (100 J/cm^2, 100 mW/cm^2) on Day 0

†Adjusted P value of comparison between days post treatment-matched mean values in PS-PDT group versus other treatment groups. Multiple unpaired (two-sample) t-tests using Holm-Šídák method correction for multiple comparisons and α = 0.05. '-' denotes comparison not performed or omitted.

Supplementary Table 10. Antitumour treatment responses in subcutaneous syngeneic MOC22 tumour models following treatment on Day 0.

**Data type**			*Tumour volume (mm^3)*				*Fold-change in tumour volume*			
**Treatment condition**	**Days post treatment**	**N**	**Mean**	**Median**	**S.D.**	**P value†**	**Mean**	**Median**	**S.D.**	**P value†**
PS-PDT	0	11	58.7	58.7	15.5	-	1.00	1.00	0.00	-
	3	11	43.0	48.0	25.0	-	0.73	0.74	0.44	-
	7	11	3.9	0.0	12.8	-	0.05	0.00	0.15	-
	14	11	3.9	0.0	12.8	-	0.05	0.00	0.15	-
Untreated control	0	13	43.0	47.1	24.7	0.083	1.00	1.00	0.00	-
	3	13	105.0	87.5	40.9	<0.001	4.18	1.50	3.70	0.01
	7	13	123.1	79.2	91.2	<0.001	5.95	1.37	6.54	0.01
	14	13	212.4	220.1	168.2	0.001	9.43	10.4	8.53	0.004

Description of treatment conditions:

PS-PDT – Tumour treated with PS-PDT (10 mg/kg, 24 hour DLI, 100 J/cm^2, 100 mW/cm^2) on Day 0

Untreated control – Untreated tumour

†Adjusted P value of comparison between days post treatment-matched mean values in PS-PDT group versus untreated control group. Multiple unpaired (two-sample) t-tests using Holm-Šídák method correction for multiple comparisons and α = 0.05. '-' denotes comparison not performed or omitted.

Supplementary Table 11. Antitumour treatment responses in orthotopic VX-2 rabbit tumour models following start of treatment on Week 0.

**Data type**			*Tumour volume (cm^3)*				*Fold-change in tumour volume*			
**Treatment condition**	**Weeks post start of treatment**	**N**	**Mean**	**Median**	**S.D.**	**P**[†]	**Mean**	**Median**	**S.D.**	**P**[†]
Single PS-PDT	0	5	0.911	0.874	0.266	0.739	1.000	1.000	0.000	-
	1	5	0.509	0.232	0.496	0.180	0.481	0.265	0.404	0.101
	2	5	0.315	0.129	0.345	0.490	0.287	0.173	0.277	0.158
	3	5	0.477	0.027	0.650	0.951	0.418	0.036	0.572	0.914
	4	5	1.113	0.005	1.626	0.650	0.972	0.006	1.457	0.515
	5	5	1.413	0.000	1.935	0.619	1.211	0.000	1.667	0.515
	6	3	0.000	0.000	0.000	-	0.000	0.000	0.000	-
Repeat PS-PDT	0	4	1.088	1.050	0.555	-	1.000	1.000	0.000	-
	1	4	1.443	1.517	0.558	-	1.448	1.176	0.579	-
	2	4	0.962	0.789	0.748	-	0.845	0.880	0.399	-
	3	4	0.505	0.223	0.649	-	0.381	0.223	0.342	-
	4	4	0.171	0.058	0.255	-	0.117	0.051	0.144	-
	5	4	0.071	0.044	0.087	-	0.056	0.044	0.058	-
	6	4	0.000	0.000	0.000	-	0.000	0.000	0.000	-
Drug control	0	3	0.260	0.246	0.151	0.111	1.000	1.000	0.000	-
	1	3	1.514	1.185	0.839	0.898	6.102	5.911	1.383	0.008
	2	3	2.704	2.468	0.893	0.107	15.38	10.05	14.14	0.087
	3	3	3.844	3.831	1.342	0.034	19.92	21.15	13.34	0.084
	4	3	5.121	4.506	2.422	0.034	25.80	31.72	16.33	0.084
	5	3	5.443	5.236	1.382	0.003	27.58	28.17	17.28	0.084
	6	-	-	-	-	-	-	-	-	-
Light control	0	3	0.538	0.498	0.294	0.335	1.000	1.000	0.000	-
	1	3	1.525	1.202	0.949	0.890	4.251	1.567	4.782	0.284
	2	3	3.960	4.095	1.087	0.022	9.732	5.643	7.809	0.180
	3	3	4.369	4.263	1.343	0.015	9.674	6.778	5.539	0.087
	4	3	5.687	5.814	1.935	0.010	14.24	7.407	12.33	0.180
	5	3	6.493	5.976	1.001	0.001	15.863	11.75	11.46	0.135
	6	-	-	-	-	-	-	-	-	-

Description of treatment conditions:

Single PS-PDT – Tumour treated once with PS-PDT (10 mg/kg, 24 hour DLI, 200 J total) on Week 0

**Supplementary Table 11.** Antitumour treatment responses in orthotopic VX-2 rabbit tumour models following start of treatment on Week 0.
Repeat PS-PDT – Tumour treated weekly with PS-PDT (10 mg/kg, 24 hour DLI, 200 J total) on Weeks 0, 1, and 2<br>*Drug control* – Rabbit administered PS only (10 mg/kg) on Week 0<br>*Light control* – Rabbit administered "two-step" PDT light treatment only (200 J total) on Week 0<br>†Adjusted P value of comparison between weeks post treatment-matched mean values in Repeat PS-PDT group versus other treatment groups. Multiple unpaired (two-sample) t-tests using Holm-Šídák method correction for multiple comparisons and $\alpha = 0.05$. '-' denotes comparison not performed or omitted.

Supplementary Table 12. Histopathological reporting of individual tissue findings in healthy male New Zealand white rabbits administered PS nanoparticles (30 mg/kg, IV) and recovered for 28-days. Evaluations performed unblinded by a board-certified veterinary pathologist on single H&E-stained level for each tissue.

**Tissues**	**Individual Findings**					**Total Abnormal Findings**	**Mean Score**	**Descriptions/Notes**
	**#1**	**#2**	**#3**	**#4**	**#5**			
Heart	0	0	0	0	1	1/5	0.2	#5: Myofibre hypereosinophilia, likely postmortem artefact
Lungs	0	0	0	0	0	0/5	0.0	No significant findings
Kidney	0	0	0	0	0	0/5	0.0	No significant findings
Liver	0	0	2	2	1	3/5	1.0	#3, #4, #5: Hepatocyte with fine glycogen type vacuolation and some micovesicular vacuolation in zonal, centrilobular, or all regions, likely background changes
Spleen	0	0	0	0	0	0/5	0.0	No significant findings
Small intestine	0	0	0	0	0	0/5	0.0	No significant findings
Large intestine	0	0	0	0	0	0/5	0.0	No significant findings
Brain	0	2	0	0	0	1/5	0.4	#2: Prominent vacuolation throughout the white matter of cerebellum and brain stem, likely postmortem artefact
Eyeball (globe)	0	0	0	0	0	0/5	0.0	No significant findings

Changes observed in organs were graded on scale of: None = 0; Minimal = 1; Mild = 2; Moderate = 3; Marked = 4; Severe = 5.
The following criteria were used for scoring:

0 - None indicated tissues were considered to be within normal limits, under the conditions of the study.

1 - Minimal lesions were those that appeared to be the least detectable within reasonable viewing.

2 - Mild lesions were easily seen, but enveloped a small amount of tissue area (up to 10%).

3 - Moderate lesions were prominent and may have involved from 10% to 50% of the potential area.

4 - Marked lesions were extensive and may have involved from 50% to 75% of available area.

5 - Severe lesions were interpreted as within the worst possible scenario.

SUPPLEMENTARY TABLES REFERENCES

1. Perrault SD, Walkey C, Jennings T, Fischer HC, Chan WCW. Mediating Tumor Targeting Efficiency of Nanoparticles Through Design. Nano Lett. 2009;9:1909–15.
2. Lee H, Fonge H, Hoang B, Reilly RM, Allen C. The Effects of Particle Size and Molecular Targeting on the Intratumoral and Subcellular Distribution of Polymeric Nanoparticles. Mol Pharmaceutics. 2010;7:1195–208.
3. Sykes EA, Chen J, Zheng G, Chan WCW. Investigating the Impact of Nanoparticle Size on Active and Passive Tumor Targeting Efficiency. ACS Nano. 2014;8:5696–706.
4. Dai Q, Wilhelm S, Ding D, Syed AM, Sindhwani S, Zhang Y, et al. Quantifying the Ligand-Coated Nanoparticle Delivery to Cancer Cells in Solid Tumors. ACS Nano. 2018;12:8423–35.
5. Kingston BR, Syed AM, Ngai J, Sindhwani S, Chan WCW. Assessing micrometastases as a target for nanoparticles using 3D microscopy and machine learning. Proc Natl Acad Sci USA. 2019;116:14937–46.
6. Ouyang B, Poon W, Zhang Y-N, Lin ZP, Kingston BR, Tavares AJ, et al. The dose threshold for nanoparticle tumour delivery. Nat Mater. 2020;19:1362–71.
7. Gross MW, Karbach U, Groebe K, Franko AJ, Mueller-Klieser W. Calibration of misonidazole labeling by simultaneous measurement of oxygen tension and labeling density in multicellular spheroids. Int J Cancer. 1995;61:567–73.
8. Kizaka-Kondoh S, Konse-Nagasawa H. Significance of nitroimidazole compounds and hypoxia-inducible factor-1 for imaging tumor hypoxia. Cancer Science. 2009;100:1366–73.
9. Fenton BM, Paoni SF, Beauchamp BK, Ding I. Zonal image analysis of tumour vascular perfusion, hypoxia, and necrosis. Br J Cancer. 2002;86:1831–6.
10. Zaidi M, Fu F, Cojocari D, McKee TD, Wouters BG. Quantitative Visualization of Hypoxia and Proliferation Gradients Within Histological Tissue Sections. Front Bioeng Biotechnol. 2019;7:397.
11. The Jackson Laboratory. Physiological Data Summary – C57BL/6J (000664) [Internet]. Available from: http://jackson.jax.org/rs/444-BUH-304/images/physiological_data_000664.pdf
12. Zoetis Services. VETSCAN® HM5 Reference Ranges (Additional Species) [Internet]. 2021. Report No.: VTS-00426. Available from: https://www.zoetisus.com/content/_assets/docs/Diagnostics/technical-papers/HM5-Reference-Ranges-iPad-VTS-00426.pdf
13. Zoetis Services. VETSCAN® VS2 Reference Ranges (SI Units) [Internet]. 2021. Report No.: VTS-00038B R2. Available from: https://www.zoetisus.com/content/_assets/docs/Diagnostics/technical-papers/VETSCAN-VS2-Reference-Ranges-VTS-00038.pdf
