# Supplementary material for: Nanoparticle-mediated Photodynamic Therapy as a Method to Ablate Oral Cavity Squamous Cell Carcinoma in Preclinical Models
Source: Cancer Res Commun. 2024 Mar 15;4(3):796–810. doi: 10.1158/2767-9764.CRC-23-0269 (PMC10941731; doi:10.1158/2767-9764.CRC-23-0269)
Supplement: Figure S1 — Supplementary figure 1 and legend. [file crc-23-0269-s03.pdf]

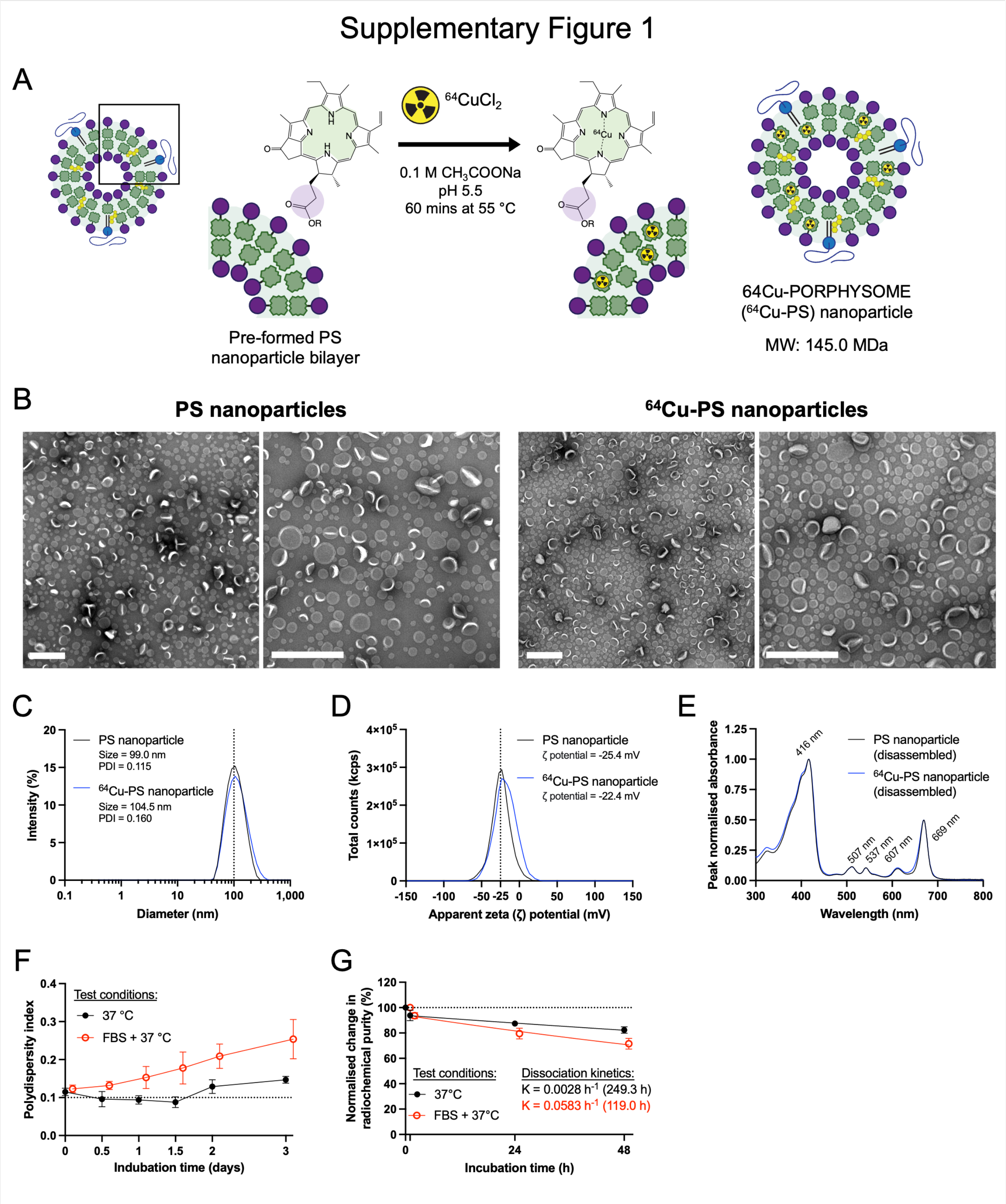

Supplementary Figure 1. Physicochemical characterisation and physiological stability studies of PS and ^{64}Cu-labelled PS nanoparticles. (A) One-step labelling procedure of pre-formed PS nanoparticles with positron-emitting copper-64 (^{64}Cu) to prepare ^{64}Cu-PS (used for pharmacokinetic and tissue distribution studies). (B) Transmission electron microscopy of negatively stained PS and ^{64}Cu-PS nanoparticle morphologies. Scale bar = 500 µm. (C) Hydrodynamic size and polydispersity index (PDI) of PS and ^{64}Cu-

PS with dynamic light scattering (DLS). Units %. Lines represents overall mean of replicates. N=6 samples/nanoparticle type. (D) Zeta potential of PS and ^{64}Cu-PS in distilled water with phase analysis light scatting (PALS). Lines represents overall mean of replicates. N=6 samples/nanoparticle type. (E) Peak normalised UV/Vis absorbance spectra of intact and disassembled PS and ^{64}Cu-PS nanoparticles (concentration matched) in aqueous media. Intact PS and ^{64}Cu-PS are suspended in 1x PBS and disassembled PS are treated with a non-ionic surfactant (Triton X-100). Absolute and local absorbance maxima wavelengths are labelled for each particle structure type. (F) Size stability of PS nanoparticles assayed by DLS at 37 °C and with 50 v/v% foetal bovine serum (FBS) to simulate physiological conditions. Increased PDI values correspond with broadening of the nanoparticle size distribution. Unitless. Mean ± standard deviation. N=3~4 samples/test condition. (G) Chelation stability of ^{64}Cu-PS nanoparticles assayed by radiochemical purity at 37 °C and with 50 v/v% FBS to simulate physiological conditions. Dissociation kinetics for each test condition fit using 1–48 h data with a mono-exponential decay model. Units %. Mean ± standard deviation. N=5 samples/test condition.
