# Supplementary material for: Nanoparticle-mediated Photodynamic Therapy as a Method to Ablate Oral Cavity Squamous Cell Carcinoma in Preclinical Models
Source: Cancer Res Commun. 2024 Mar 15;4(3):796–810. doi: 10.1158/2767-9764.CRC-23-0269 (PMC10941731; doi:10.1158/2767-9764.CRC-23-0269)
Supplement: Figure S2 — Supplementary figure 2 and legend. [file crc-23-0269-s04.pdf]

Supplementary Figure 2

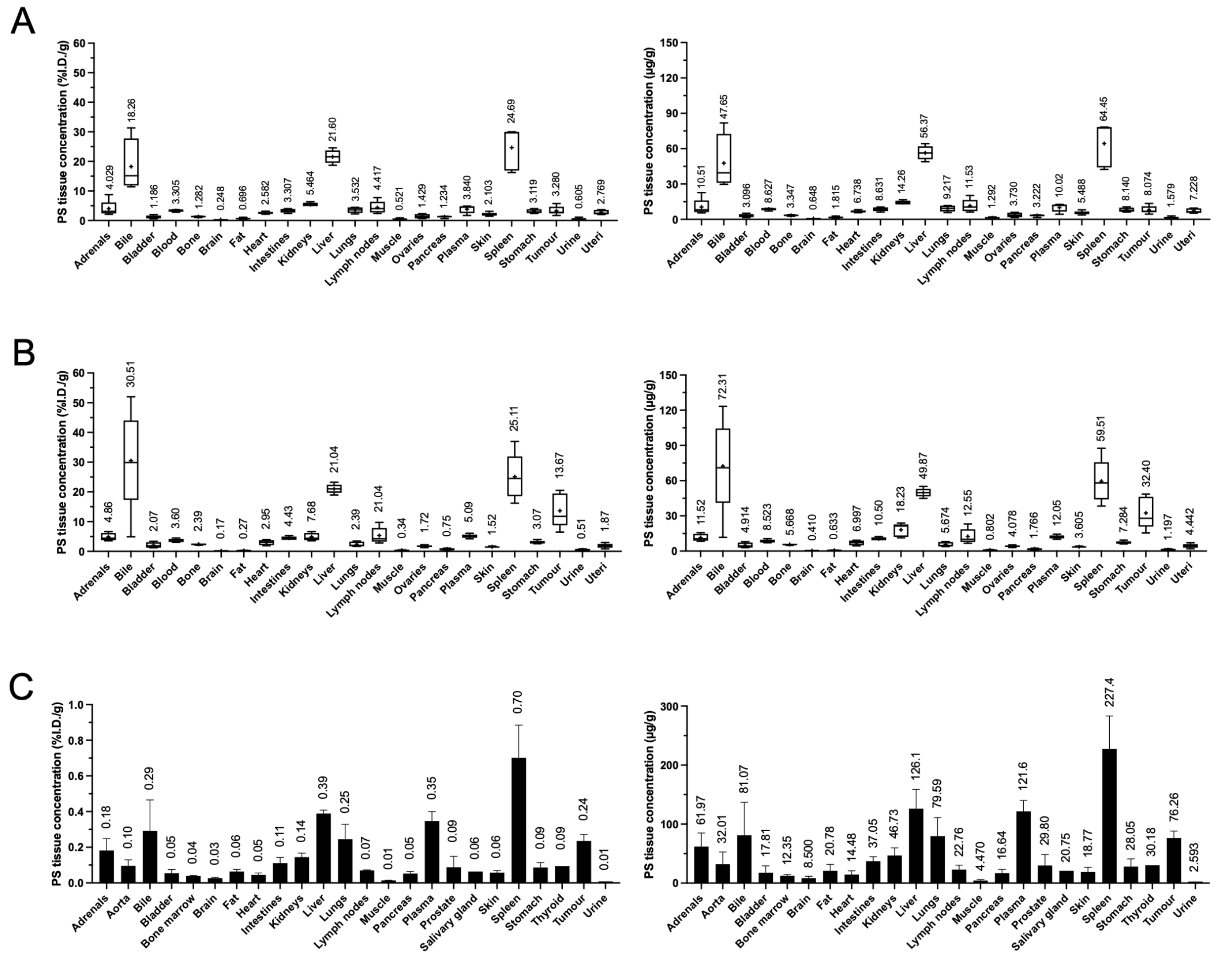

Supplementary Figure 2. Tissue concentration of PS nanoparticles in the major organs and tissues of tumour-bearing animal models of oral cavity cancer 24 hours post-injection. Units %I.D./g (left) and µg/g (right). PS (10 mg/kg, 400-500 MBq ^{64}Cu/kg, IV) tissue distribution in (A) Cal-33 xenograft tumour models and (B) syngeneic MOC22 tumour models. N=5~10 mice/tissue/model. (C) PS (10 mg/kg, IV) tissue distribution in orthotopic VX-2 rabbit tumour models. N=3 rabbits/tissue. For (A, B): Tukey box-and-whisker plot with "+" denoting mean (mean labelled above bar). Note for ^{64}Cu-based pharmacokinetic measurements, %I.D. has been radioactive decay-corrected from time of tissue measurement to the time of administration. For (C): Bar plot with mean + standard deviation (mean labelled above bar). Additional statistical summaries of PS tissue pharmacokinetics provided in **Supplementary Table 3** (Cal-33 model), **Supplementary Table 4** (MOC22 model), and **Supplementary Table 5** (VX-2 model).
