# Supplementary material for: Nanoparticle-mediated Photodynamic Therapy as a Method to Ablate Oral Cavity Squamous Cell Carcinoma in Preclinical Models
Source: Cancer Res Commun. 2024 Mar 15;4(3):796–810. doi: 10.1158/2767-9764.CRC-23-0269 (PMC10941731; doi:10.1158/2767-9764.CRC-23-0269)
Supplement: Figure S3 — Supplementary figure 3. [file crc-23-0269-s05.pdf]

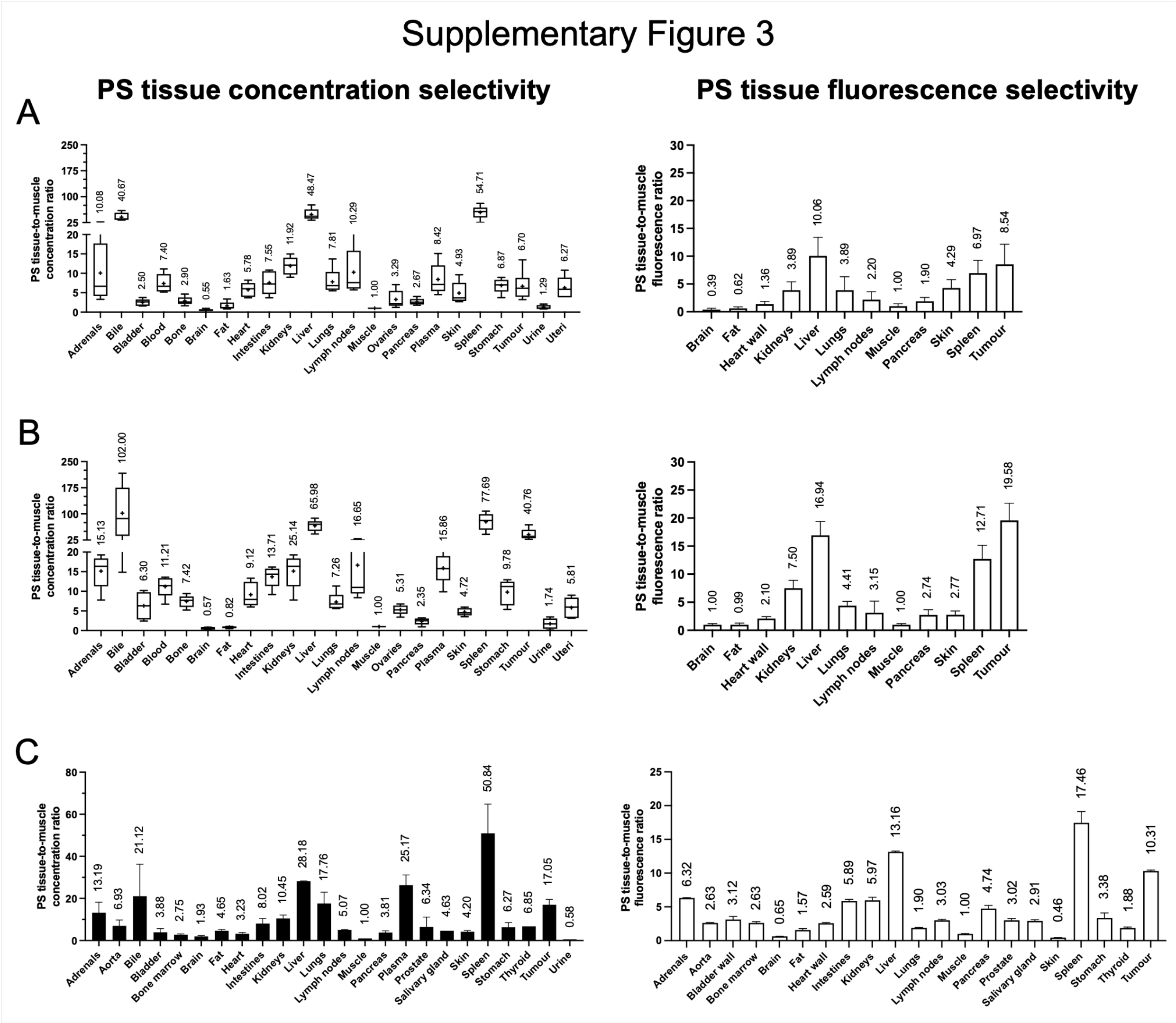

Supplementary Figure 3. Tissue selectivity of PS nanoparticles based on concentration (left) and ex vivo fluorescence signal (right) measurements in tumour-bearing animal models of oral cavity cancer 24 hours post-injection. Tissue-to-muscle ratios of PS (10 mg/kg, 400-500 MBq ^{64}Cu/kg, IV) in (A) Cal-33 xenograft tumour models and (B) syngeneic MOC22 tumour models. Unitless. N=5~10 mice/tissue/model. (C) Tissue-to-muscle ratios of PS (10 mg/kg, IV) in orthotopic VX-2 rabbit tumour models. Unitless. N=3 rabbits/tissue. For (A, B): Tukey box-and-whisker plot with "+" denoting mean (mean labelled above bar). For (A, B, C): Bar plot with mean + standard deviation (mean labelled above bar). Additional statistical summaries of PS tissue selectivity provided in **Supplementary Table 6** (concentration selectivity) and **Supplementary Table 7** (fluorescence selectivity).
