# Supplementary material for: Nanoparticle-mediated Photodynamic Therapy as a Method to Ablate Oral Cavity Squamous Cell Carcinoma in Preclinical Models
Source: Cancer Res Commun. 2024 Mar 15;4(3):796–810. doi: 10.1158/2767-9764.CRC-23-0269 (PMC10941731; doi:10.1158/2767-9764.CRC-23-0269)
Supplement: Figure S4 — Supplementary figure 4 and legend. [file crc-23-0269-s06.pdf]

Supplementary Figure 4

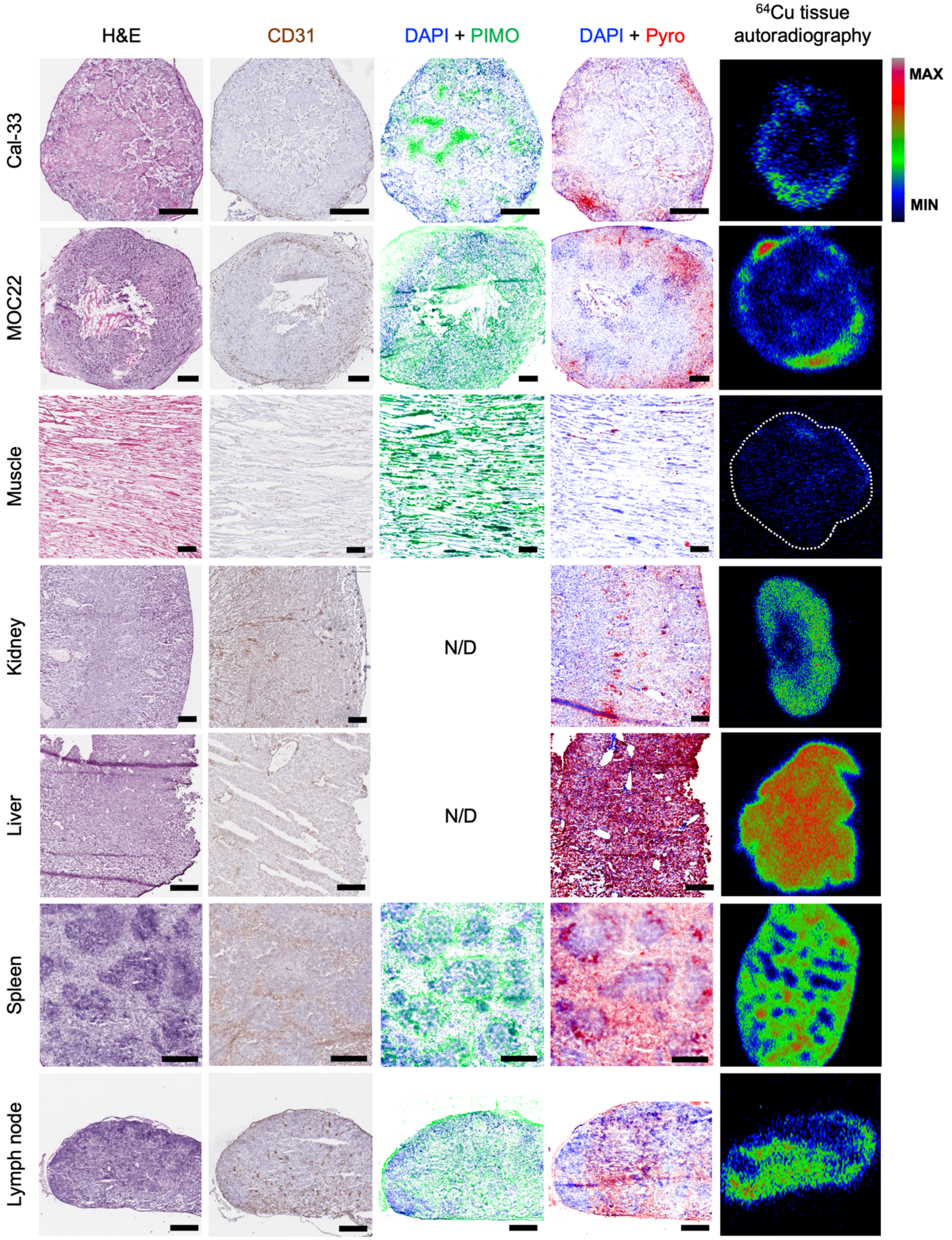

Supplementary Figure 4. Representative histology images of the major organs and tumour tissues from tumour-bearing mouse models of oral cavity cancer. PS nanoparticles (10 mg/kg, 400-500 MBq ^{64}Cu/kg, IV) were administered 24 hours prior to necropsy and excised tissues were flash frozen in optimal cutting temperature compound. Scale bars = 400 µm throughout. Abbreviations: H&E (Haematoxylin and eosin) – tissue microanatomy and cellular morphology; CD31 (Cluster of differentiation 31) – vascular/endothelial cell marker indicating blood vessels; PIMO (Pimonidazole) – fluorescent marker for tissue hypoxia; DAPI (4′,6-diamidino-2-phenylindole) –fluorescent marker for nuclear DNA; Pyro – fluorescent marker for PS nanoparticle accumulation; ^{64}Cu tissue autoradiography – intensity marker for ^{64}Cu-labelled PS radioactivity on a phosphor screen; N/D – not done.
