# Supplementary material for: Nanoparticle-mediated Photodynamic Therapy as a Method to Ablate Oral Cavity Squamous Cell Carcinoma in Preclinical Models
Source: Cancer Res Commun. 2024 Mar 15;4(3):796–810. doi: 10.1158/2767-9764.CRC-23-0269 (PMC10941731; doi:10.1158/2767-9764.CRC-23-0269)
Supplement: Figure S5 — Supplementary figure 5 and legend. [file crc-23-0269-s07.pdf]

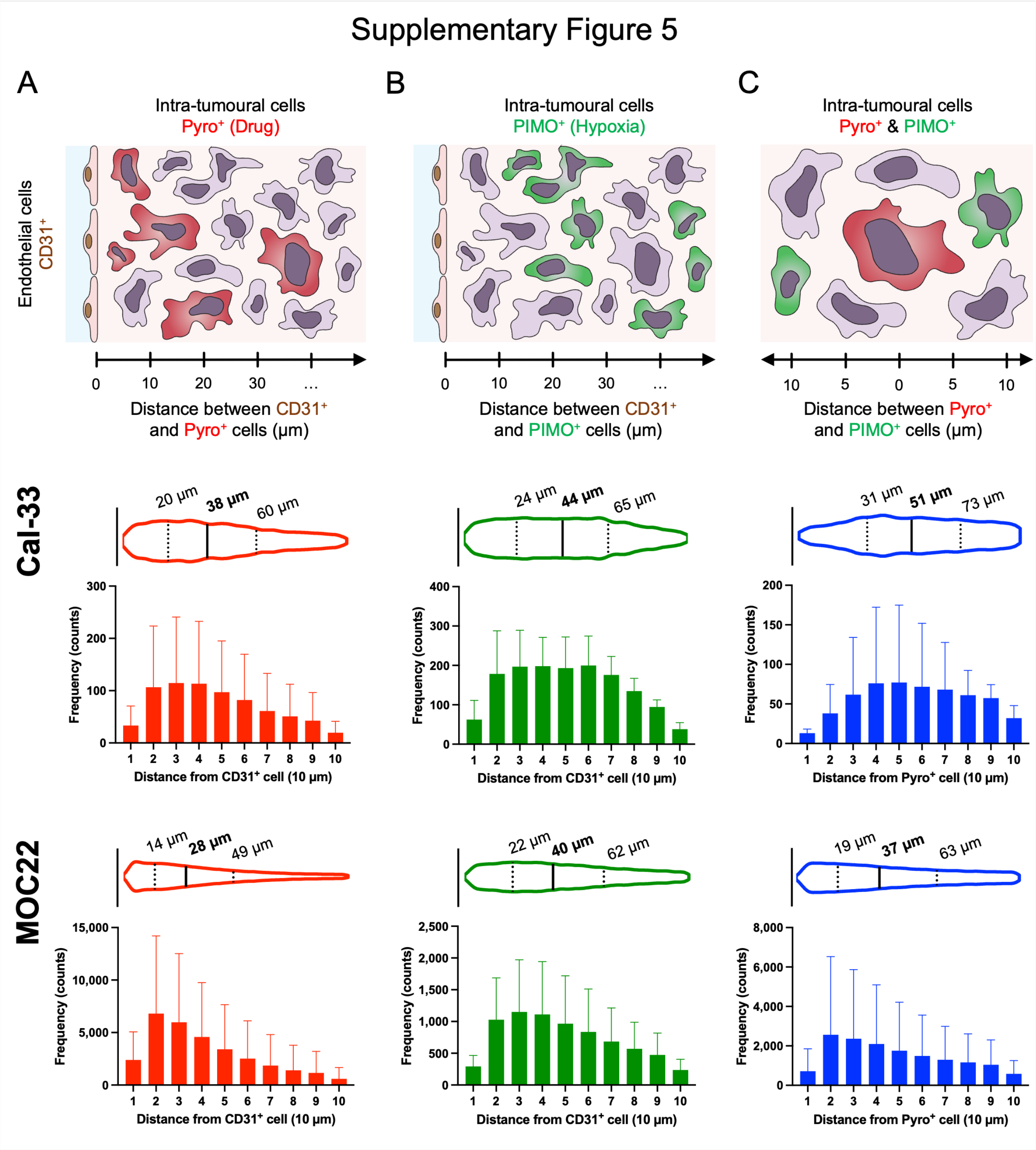

Supplementary Figure 5. Quantitative spatial analysis of the intratumoural distribution of chromogenic and fluorescent markers in Cal-33 and MOC22 tumour histology. Analysis schematic and proximity distributions for (A) Pyro$^+$ cell distances to CD31$^+$ cells (CD31←Pyro), (B) PIMO$^+$ cell distances to CD31$^+$ cells (CD31←PIMO), and (C) PIMO$^+$ cell distances to Pyro$^+$ cells (Pyro←PIMO) in Cal-33 (middle row) and MOC22 (bottom row) tumours. Abbreviations: CD31 (Cluster of differentiation 31), vascular/endothelial cell marker indicating blood vessels; PIMO (Pimonidazole), fluorescent marker for tissue hypoxia; Pyro, fluorescent marker for PS nanoparticle accumulation. Units µm. N = 4 Cal-33

tumours, 5 MOC22 tumours. In density plots: Q1 and Q3 distances identified by dotted lines, and median distance (in bold) identified with solid line. In frequency histograms (bar plots): mean + S.D. Statistical testing was not performed.

Interpretation for Supplementary Figure 5

In panel A we observed that PS nanoparticles (Pyro$^+$ maker) accumulated in cells proximal to tumour blood vessels (CD31$^+$ marker). In Cal-33 tumours, 50% of Pyro$^+$ cells were spatially distributed within 38 μm of CD31$^+$ cells, corresponding to ~2.5x cell widths (average max calliper distance of Cal-33 tumour cell 15.3 ± 3.7 μm). In MOC22 tumours, 50% of Pyro$^+$ cells were spatially distributed within 28 μm of CD31$^+$ cells, corresponding to ~2.0x cell widths (average max calliper distance of MOC22 tumour cell 15.4 ± 3.8 μm). These observations are consistent with literature reported penetration distances for other physicochemically similar nanoparticles in tumour models (1–6). Thus, we interpret these results as suggesting PS nanoparticle's penetration into tumours was comparatively restricted to 1~2 cells from tumour-associated blood vessels. However, an important caveat to this interpretation is that the analysis does not differentiate whether all CD31$^+$ blood vessels are adequately perfused with blood; in fact, observing the near complete lack of PS nanoparticle signal from vessels in the centre/core of the tumour in **Figure 4A,B** and **Supplementary Figure 4** suggests these vessels are likely non-functioning. Also notice in panel A the significant difference in the number of Pyro$^+$ cells detected in the Cal-33 tumours (~1,000 cells per tumour cross-section) versus in the MOC22 tumours (~10,000 cells per tumour cross-section); this observation is consistent with the reported divergence in PS accumulation between the two tumour models (see **Figure 3C**).

Panel B exhibits the intratumoural distribution of hypoxia marker pimonidazole (PIMO$^+$ marker). Pimonidazole is a prodrug that forms covalent bonds to cellular macromolecules at oxygen levels below 10 mmHg and visualises poorly oxygenated regions in histological sections of tumours (7,8). In both Cal-33 and MOC22 tumours there was an approximately uniform (or linear) increase in the number of hypoxic PIMO$^+$ cells with increasing distances from tumour-associated CD31$^+$ blood vessels, a pattern that is consistent with other literature reports in tumours (9,10). Expressing the number of hypoxic PIMO$^+$ cells as a percentage of the total number of viable nucleated cells in the tumour cross-section, we observed that 6.8 ± 1.4% and 9.8 ± 7.0% of cells in Cal-33 and MOC22 tumours, respectively, were severely hypoxic (see Figure 4C). Given the critical importance of molecular oxygen for the mechanism of action of type II photosensitiser drug such as PS, these data suggest that both tumour types should be readily amendable to PDT treatments.

Lastly in panel C we compared the spatial distribution of hypoxic cells with pimonidazole signal (PIMO$^+$ marker) in relation to cells with PS nanoparticle signal (Pyro$^+$ marker) (i.e., Pyro←PIMO). As was

demonstrated in **Figure 4A,B** and **Supplementary Figure 4**, the spatial distribution of $PIMO^+$ cells and $Pyro^+$ cells in both tumour models do not necessarily overlap with one another. Considering that the primary pathway for systemically administered PS to accumulate in the tumour is via perfused blood vessels, it is intuitive that these same microregions of the tumour should also be receiving sufficient oxygenation from the vascular supply. In panel C we observed that this was indeed the case with the median distances of $PIMO^+$ cells from $Pyro^+$ cells equal to 51 µm (~3.4x cell widths) in Cal-33 tumours and to 37 µm (~2.5x cell widths) in MOC22 tumours. We interpret these results as suggesting that PS predominantly accumulate in tumour regions and cells with comparatively normoxic conditions (>10 mmHg O_2); an important finding given the necessity of sufficient O_2 for ensuring efficient generation of cytotoxic reactive oxygen species during PS-PDT.

There are several notable limitations with quantitative spatial analysis of histology slides such as we have performed here: firstly, because of incompatibilities between the staining protocols for CD31 (chromogenic), PIMO and Pyro (fluorescent) markers, the proximity analysis for comparing the spatial distribution of these markers was performed across separate histology slides from the same tumour. Digitised images of brightfield and fluorescent slides were co-registered to one another using a combination of algorithmic and manual approaches; nevertheless, even small differences in preparations between slides, registration artefacts, etc. can amplify inaccuracies in the reported spatial measurements. Secondly, the algorithm for identifying the marker positivity of nucleated cells assigns only one marker per cell. In cases where cells were positive for more than one marker (e.g., $CD31^+$ tumour endothelial cells), the marker with the higher relative intensity was assigned by the algorithm. Thirdly, aside from vascular endothelial cells ($CD31^+$), the identities of the other cell types positive for PS nanoparticle uptake ($Pyro^+$) or hypoxia ($PIMO^+$) are unknown (e.g., tumour cells, tumour-associate macrophages, cancer-associated fibroblasts, etc.). And lastly, the analysis performed here is a two-dimensional cross-section of a three-dimensional tumour environment. Therefore, generalisation of the results from 2D spatial analysis performed above to a 3D tumour should be interpreted cautiously.

REFERENCES

1. Perrault SD, Walkey C, Jennings T, Fischer HC, Chan WCW. Mediating Tumor Targeting Efficiency of Nanoparticles Through Design. Nano Lett. 2009;9:1909–15.
2. Lee H, Fonge H, Hoang B, Reilly RM, Allen C. The Effects of Particle Size and Molecular Targeting on the Intratumoral and Subcellular Distribution of Polymeric Nanoparticles. Mol Pharmaceutics. 2010;7:1195–208.
3. Sykes EA, Chen J, Zheng G, Chan WCW. Investigating the Impact of Nanoparticle Size on Active and Passive Tumor Targeting Efficiency. ACS Nano. 2014;8:5696–706.

4. Dai Q, Wilhelm S, Ding D, Syed AM, Sindhwani S, Zhang Y, et al. Quantifying the Ligand-Coated Nanoparticle Delivery to Cancer Cells in Solid Tumors. ACS Nano. 2018;12:8423–35.
5. Kingston BR, Syed AM, Ngai J, Sindhwani S, Chan WCW. Assessing micrometastases as a target for nanoparticles using 3D microscopy and machine learning. Proc Natl Acad Sci USA. 2019;116:14937–46.
6. Ouyang B, Poon W, Zhang Y-N, Lin ZP, Kingston BR, Tavares AJ, et al. The dose threshold for nanoparticle tumour delivery. Nat Mater. 2020;19:1362–71.
7. Gross MW, Karbach U, Groebe K, Franko AJ, Mueller-Klieser W. Calibration of misonidazole labeling by simultaneous measurement of oxygen tension and labeling density in multicellular spheroids. Int J Cancer. 1995;61:567–73.
8. Kizaka-Kondoh S, Konse-Nagasawa H. Significance of nitroimidazole compounds and hypoxia-inducible factor-1 for imaging tumor hypoxia. Cancer Science. 2009;100:1366–73.
9. Fenton BM, Paoni SF, Beauchamp BK, Ding I. Zonal image analysis of tumour vascular perfusion, hypoxia, and necrosis. Br J Cancer. 2002;86:1831–6.
10. Zaidi M, Fu F, Cojocari D, McKee TD, Wouters BG. Quantitative Visualization of Hypoxia and Proliferation Gradients Within Histological Tissue Sections. Front Bioeng Biotechnol. 2019;7:397.
