# Supplementary material for: Nanoparticle-mediated Photodynamic Therapy as a Method to Ablate Oral Cavity Squamous Cell Carcinoma in Preclinical Models
Source: Cancer Res Commun. 2024 Mar 15;4(3):796–810. doi: 10.1158/2767-9764.CRC-23-0269 (PMC10941731; doi:10.1158/2767-9764.CRC-23-0269)
Supplement: Figure S6 — Supplementary figure 6 and legend. [file crc-23-0269-s08.pdf]

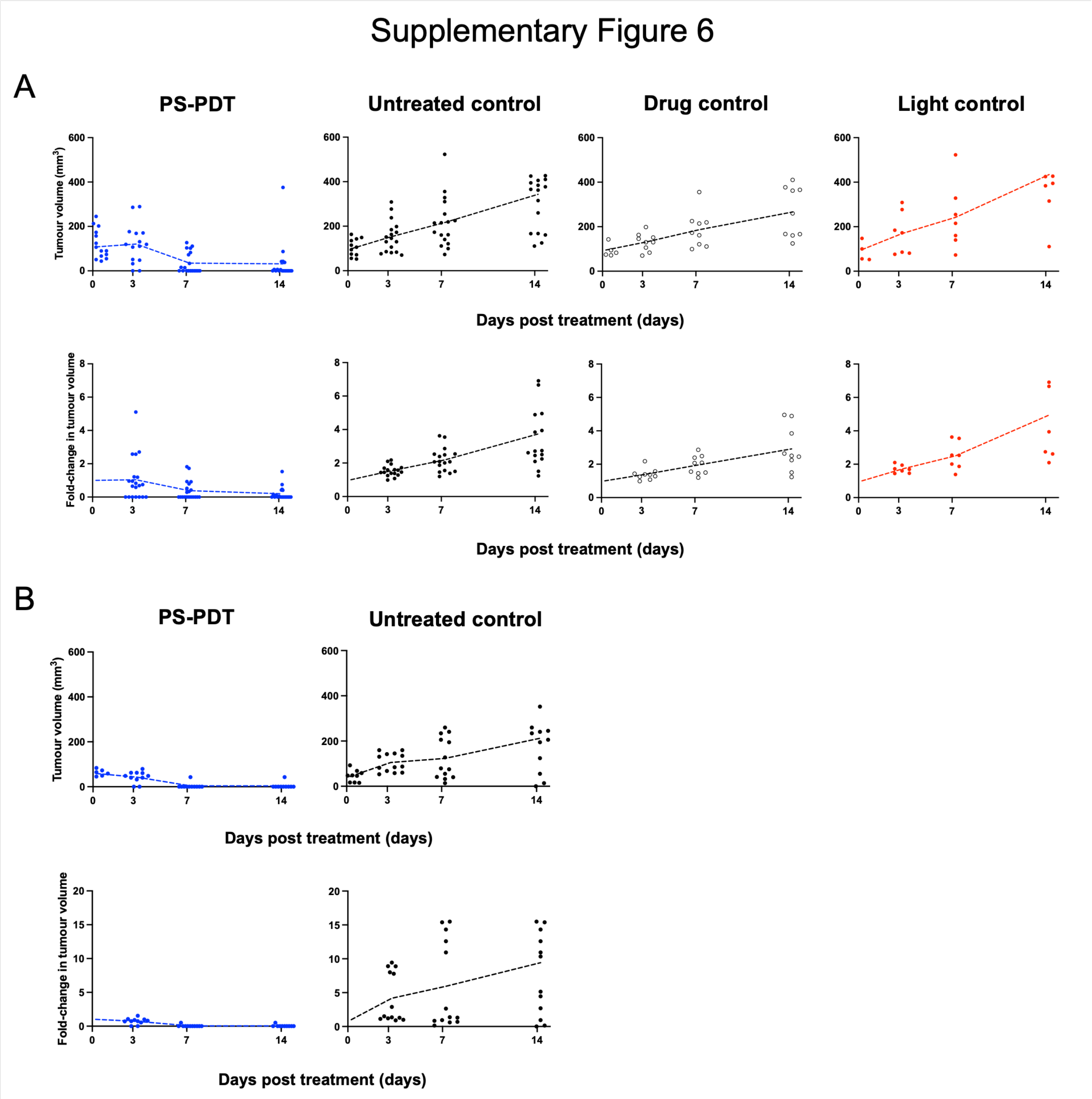

Supplementary Figure 6. Antitumour treatment response to PS nanoparticle mediated photodynamic therapy (PS-PDT) in tumour-bearing mouse models of oral cavity cancer. Treatment responses in (A) subcutaneous Cal-33 xenograft tumour models and (B) subcutaneous syngeneic MOC22 tumour models. Top rows: change in measured tumour volume (spherical volume calculated from greatest dimension measured). Bottom rows: fold-change in tumour volume from measurement on Day 0 (i.e., start of treatments). PS-PDT group treated once with surface PDT (10 mg/kg, 24 hour DLI, 100 J/cm^2, 100 mW/cm^2) on Day 0. Untreated control group received no treatments. Drug control group received PS administration only (10 mg/kg) on Day 0. Light control group received surface PDT light treatment only (100 J/cm^2, 100 mW/cm^2) on Day 0. Bullet points represent individual replicates. Dashed line represents

trend in mean value of plotted parameters. For (A): N=20 tumours (PS-PDT), 17 tumours (untreated control), 9 tumours (drug control), and 7 tumours (light control). Statistical summary provided in **Supplementary Table 9**. For (B): N=11 tumours (PS-PDT), and 13 tumours (untreated control). Statistical summary provided in **Supplementary Table 10**.
