# Supplementary material for: Nanoparticle-mediated Photodynamic Therapy as a Method to Ablate Oral Cavity Squamous Cell Carcinoma in Preclinical Models
Source: Cancer Res Commun. 2024 Mar 15;4(3):796–810. doi: 10.1158/2767-9764.CRC-23-0269 (PMC10941731; doi:10.1158/2767-9764.CRC-23-0269)
Supplement: Figure S7 — Supplementary figure 7 and legend. [file crc-23-0269-s09.pdf]

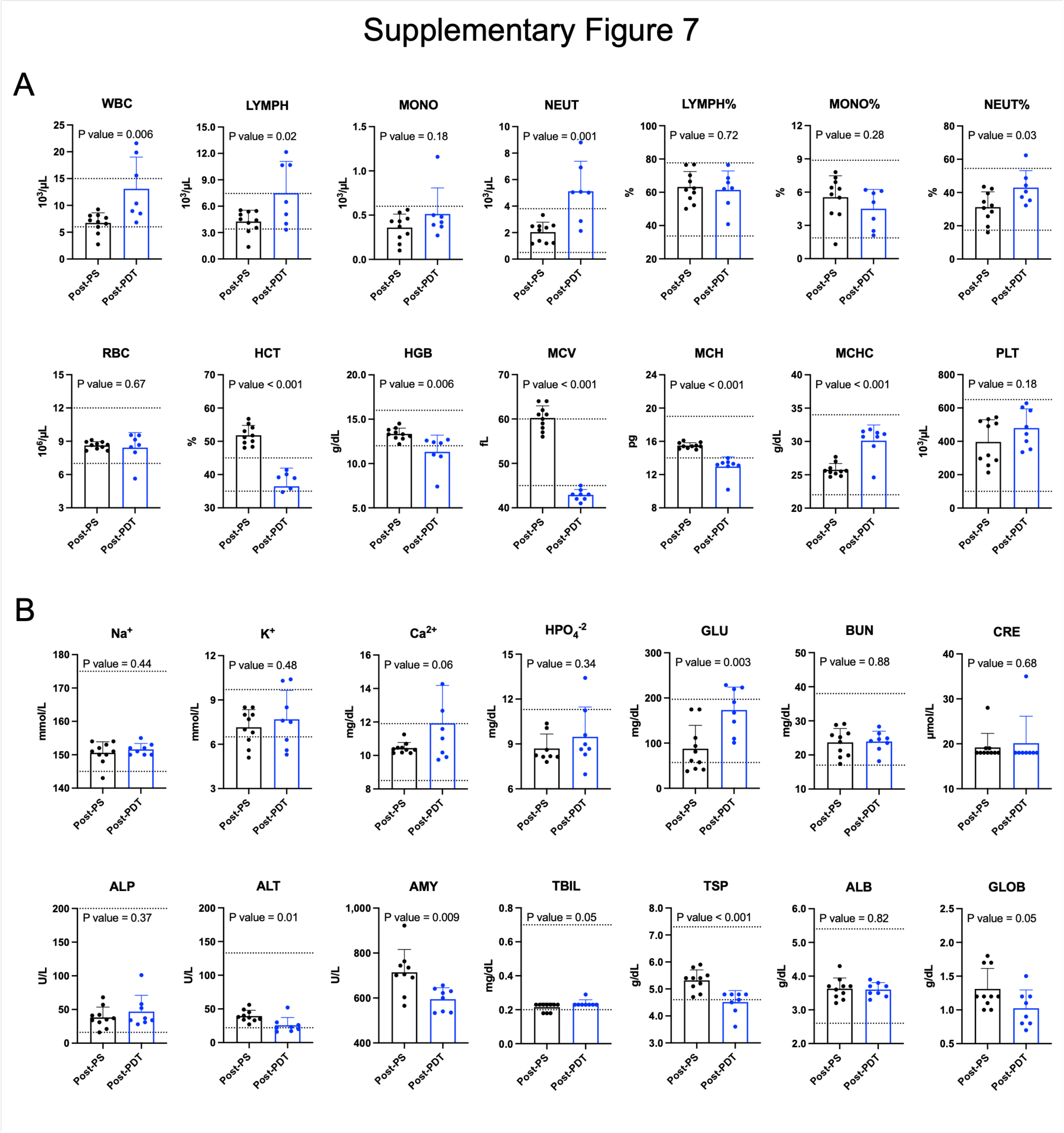

Supplementary Figure 7. Clinical pathology in PS-PDT treated immunocompetent C57BL/6 WT mice with subcutaneous syngeneic MOC22 tumours. Blood samples collected in terminal procedures at (i) 24 hours post-PS nanoparticle (10 mg/kg, IV) injection ("Post-PS"), and at (ii) 72 hours post-PS-PDT ("Post-PDT") (100 J/cm^2, 100 mW/cm^2). (A) Full blood count and (B) clinical biochemistry. Bar plot with mean + standard deviation. ● represent individual replicates. Normal limits for each parameter represented by dotted lines (1). N = 10 Post-PS mice, and 8 Post-PDT mice. Statistics: unpaired t-tests without correction for multiple comparisons and α = 0.05. Abbreviations (units): WBC, white blood cells (×10^3 cells/µL); LYMPH, lymphocytes (×10^3 cells/µL); MONO, monocytes (×10^3 cells/µL); NEU, neutrophil count (×10^3

cells/μL); RBC, red blood cells (×10^6 cells/μL); HGB, haemoglobin (g/dL); HCT, haematocrit; MCV, mean corpuscular volume (fL); PLT, platelets (×10^3/μL); MPV, mean platelet volume (fL); Na^+, sodium (mmol/L); K^+, potassium (mmol/L), Ca^{2+}, calcium (mg/dL), HPO_4^{-2}, phosphorous (mg/dL); GLU, blood glucose (mg/dL); BUN, blood urea nitrogen (mg/dL); CRE, creatinine (μmol/L); ALP, alkaline phosphatase (U/L); ALT, alanine aminotransferase (U/L); AMY, amylase (U/L); TBIL, total bilirubin (mg/dL); TSP, total serum protein (g/dL); ALB, albumin (g/dL); GLOB, globulin (g/dL).

REFERENCES

1. The Jackson Laboratory. Physiological Data Summary – C57BL/6J (000664) [Internet]. Available from: http://jackson.jax.org/rs/444-BUH-304/images/physiological_data_000664.pdf
