# Supplementary material for: Nanoparticle-mediated Photodynamic Therapy as a Method to Ablate Oral Cavity Squamous Cell Carcinoma in Preclinical Models
Source: Cancer Res Commun. 2024 Mar 15;4(3):796–810. doi: 10.1158/2767-9764.CRC-23-0269 (PMC10941731; doi:10.1158/2767-9764.CRC-23-0269)
Supplement: Figure S8 — Supplementary figure 8 and legend. [file crc-23-0269-s10.pdf]

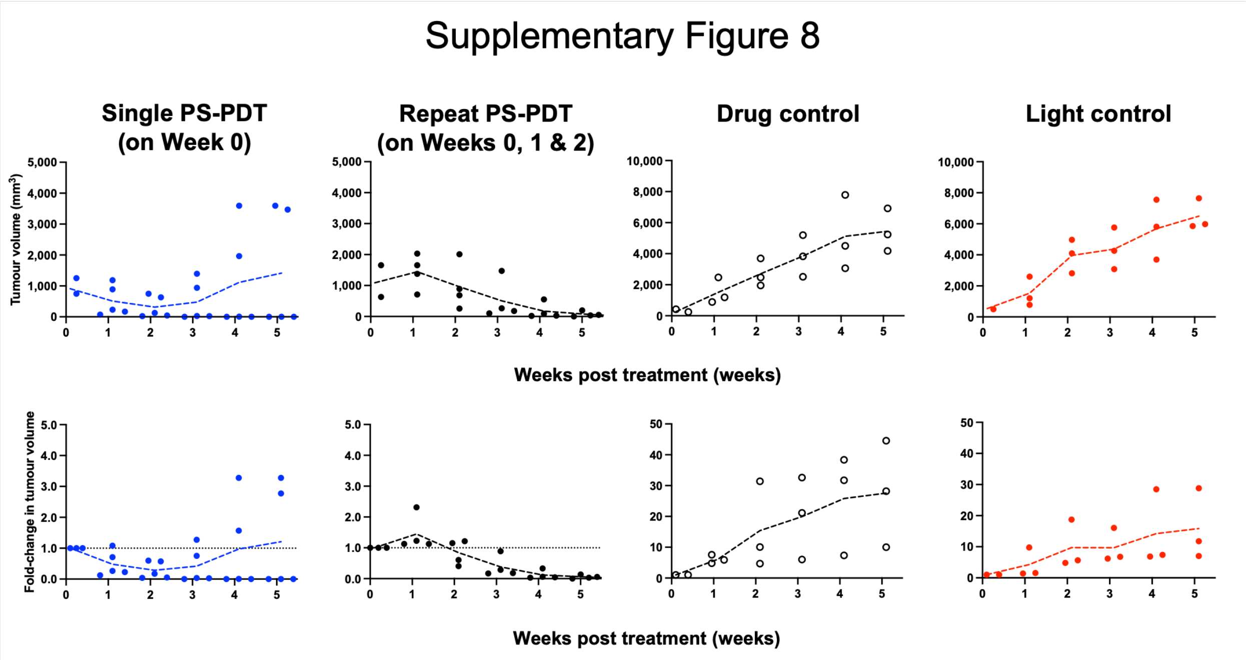

Supplementary Figure 8. Antitumour treatment response to PS nanoparticle mediated photodynamic therapy (PS-PDT) in orthotopic VX-2 rabbit tumour models. Top row: change in measured tumour volume (volume calculated from direct measurements on diagnostic CT imaging). Bottom row: fold-change in tumour volume from measurement on Week 0 (i.e., start of treatments). PS-PDT treatment (10 mg/kg, 24 hour DLI, 200 J total) was performed either once on Week 0 (Single PS-PDT group) or repeated weekly on Weeks 0, 1, and 2 (Repeat PS-PDT group) for a total of three treatments in the same tumour (600 J total/tumour). Drug control group administered PS only (10 mg/kg) on Week 0. Light control group administered "two-step" PDT light treatment only (200 J total) on Week 0. Bullet points represent individual replicates. N=5 tumours (Single PS-PDT), 4 tumours (Repeat PS-PDT), 3 tumours (Drug control), and 3 tumours (Light control). Dashed line represents trend in mean value of plotted parameters. Statistical summary provided in **Supplementary Table 11**.
