# Supplementary material for: Nanoparticle-mediated Photodynamic Therapy as a Method to Ablate Oral Cavity Squamous Cell Carcinoma in Preclinical Models
Source: Cancer Res Commun. 2024 Mar 15;4(3):796–810. doi: 10.1158/2767-9764.CRC-23-0269 (PMC10941731; doi:10.1158/2767-9764.CRC-23-0269)
Supplement: Figure S9 — Supplementary figure 9 and legend. [file crc-23-0269-s11.pdf]

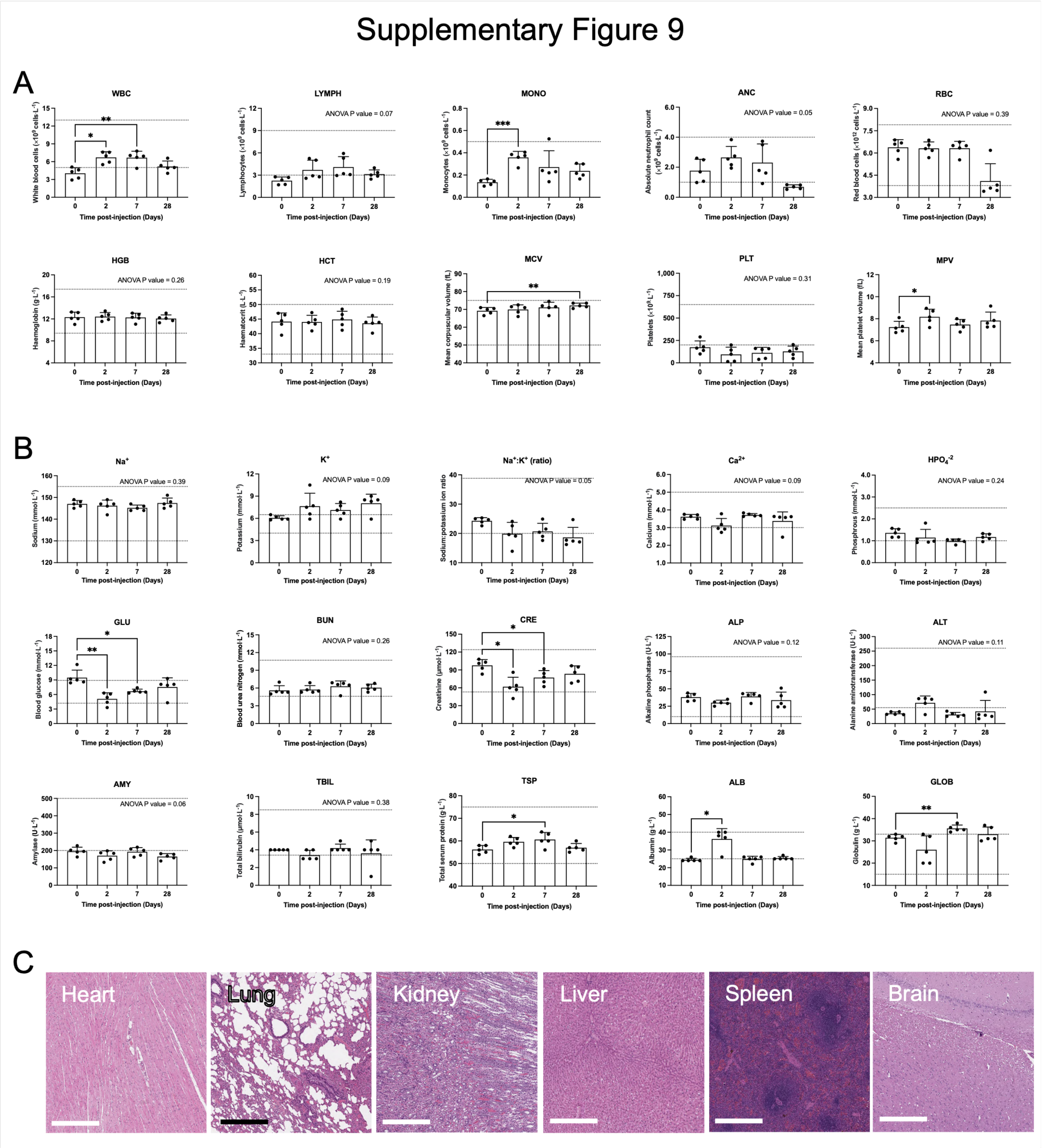

Supplementary Figure 9. Clinical and anatomical pathology of PS administration (30 mg/kg, IV) in healthy rabbits. (A) Haematology and (B) clinical biochemistry results at baseline (Day 0), and 2-, 7-, and 28-days post-PS administration. Bar plot with mean + standard deviation. ● represent individual replicates. Normal limits for each parameter represented by dotted lines (1,2). N = 5 male rabbits. Statistics: Repeat measure one-way ANOVA with Geisser-Greenhouse correction and α = 0.05. If ANOVA P value was statistically significant, a Dunnett's multiple comparison test against Day 0 timepoint (control column) was performed with α = 0.05 (only statistically significant differences identified in figures). Abbreviations

(units): WBC, white blood cells ($\times 10^9$ cells/L); LYMPH, lymphocytes ($\times 10^9$ cells/L); MONO, monocytes ($\times 10^9$ cells/L); ANC, absolute neutrophil count ($\times 10^9$ cells/L); RBC, red blood cells ($\times 10^{12}$ cells/L); HGB, haemoglobin (g/L); HCT, haematocrit; MCV, mean corpuscular volume (fL); PLT, platelets ($\times 10^9$/L); MPV, mean platelet volume (fL); Na^+, sodium (mmol/L); K^+, potassium (mmol/L); Na^+:K^+, sodium:potassium ratio; Ca^{2+}, calcium (mmol/L), HPO_4^{-2}, phosphorous (mmol/L); GLU, blood glucose (mmol/L); BUN, blood urea nitrogen (mmol/L); CRE, creatinine (mmol/L); ALP, alkaline phosphatase (U/L); ALT, alanine aminotransferase (U/L); AMY, amylase (U/L); TBIL, total bilirubin (µmol/L); TSP, total serum protein (g/L); ALB, albumin (g/L); GLOB, globulin (g/L). (C) Representative images of H&E-stained histology 28-days post-PS administration. Scale bar = 500 µm throughout. Anatomical pathology reporting presented in **Supplementary Table 12**.

REFERENCES

1. Zoetis Services. VETSCAN® HM5 Reference Ranges (Additional Species) [Internet]. 2021. Report No.: VTS-00426. Available from: https://www.zoetisus.com/content/_assets/docs/Diagnostics/technical-papers/HM5-Reference-Ranges-iPad-VTS-00426.pdf
2. Zoetis Services. VETSCAN® VS2 Reference Ranges (SI Units) [Internet]. 2021. Report No.: VTS-00038B R2. Available from: https://www.zoetisus.com/content/_assets/docs/Diagnostics/technical-papers/VETSCAN-VS2-Reference-Ranges-VTS-00038.pdf
